# Dynamic CD4+ T Cell Genetics Identifies Candidate Therapeutic Targets for Allergic Rhinitis: Mendelian Randomization with Exploratory Single-Cell Contextualization

**DOI:** 10.3390/biomedicines14092077

**Published:** 2026-09-15

**Authors:** Wei Gu, Jinglei Li, Chuan Liu, Chuan Chen, Jian Wang

**Affiliations:** 1 Department of Otolaryngology, Peking Union Medical College Hospital, Chinese Academy of Medical Sciences and Peking Union Medical College, Beijing 100730, China; ent_wei.gu@outlook.com (W.G.);; 2 CAMS Oxford Institute, Nuffield Department of Medicine, University of Oxford, Oxford OX3 7BN, UK

**Keywords:** allergic rhinitis, Mendelian randomization, CD4+ T cells, expression quantitative trait loci, single-cell RNA sequencing, immunotherapy, candidate therapeutic targets, precision medicine, biomarkers

## Abstract

**Background**: Allergic rhinitis is common and clinically heterogeneous, but the genes whose genetically regulated immune-state expression influences susceptibility remain incompletely resolved. **Methods**: We integrated dynamic CD4+ T cell eQTL data (46 activation-state profiles) with allergic rhinitis GWAS summary statistics, applying transcriptome-wide Mendelian randomization (MR), colocalization, SMR/HEIDI, metabolite screening, paired single-cell RNA-seq and TCR analysis, and computational follow-up. Colocalization support was prespecified as conditional ratio PP.H4/(PP.H3 + PP.H4) > 0.7; HEIDI at *p* > 0.05. **Results**: After MHC-region exclusion, 50,654 gene-profile pairs were tested and 129 MR-significant genes were identified. Colocalization supported 418 pairs (111 genes); 70 genes passed both SMR and HEIDI; and cross-database comparison yielded a nine-gene overlap set. In GSE200107, baseline good-versus-poor responder differences were larger than treatment-induced transcriptional change; patient-level reanalysis found no pair surviving multiple-testing correction (*n* = 4 vs. 3); these findings are hypothesis-generating. Paired TCR analysis suggested greater clonotype renewal in good responders (descriptive). Structure-guided screening prioritized unvalidated binder candidates for SLC25A46 and TLR1 (TLR1 TIR docking −5.86 kcal/mol; MD RMSD 0.20 nm). **Conclusions**: An exploratory metabolite screen yielded nominally significant indirect-effect signals, but the candidate metabolite did not colocalize with the disease locus. This framework prioritizes candidate therapeutic targets for allergic rhinitis.

## 1. Introduction

Allergic rhinitis affects 10–30% of adults in many populations and remains a major cause of chronic symptoms, sleep disturbance, impaired quality of life, and repeated medication use [1,2,3]. Standard treatment with antihistamines, intranasal corticosteroids, and allergen immunotherapy is often effective, but response is heterogeneous, and durable disease modification is still not achieved uniformly across patients [2,3,4,5].

CD4+ T cells are central to allergic inflammation because they integrate epithelial and innate signals, coordinate type 2 cytokine production, provide B-cell help, and shape immune memory and tolerance [6,7,8]. Genome-wide association studies have identified many loci shared across asthma, hay fever, and eczema, as well as allergic-rhinitis-specific risk loci, but GWAS alone rarely resolves the effector gene, the relevant immune state, or the direction of perturbation needed for target nomination [9,10,11]. This remains a major obstacle in translating association signal into mechanism and therapy.

Dynamic eQTL data offer one way to narrow this gap. The Soskic resource maps gene regulation across 46 CD4+ T cell activation-state profiles from 119 donors and captures regulatory effects that are weak or absent outside the relevant activation window [12]. This temporal layer matters in allergic disease, where early activation, effector polarization, and later remodeling are unlikely to have equivalent biological meaning. Related studies in cardiometabolic disease and colorectal cancer have already shown that the same analytical framework can prioritize disease-relevant targets from dynamic CD4+ T cell genetics [13,14].

**Figure 1:**
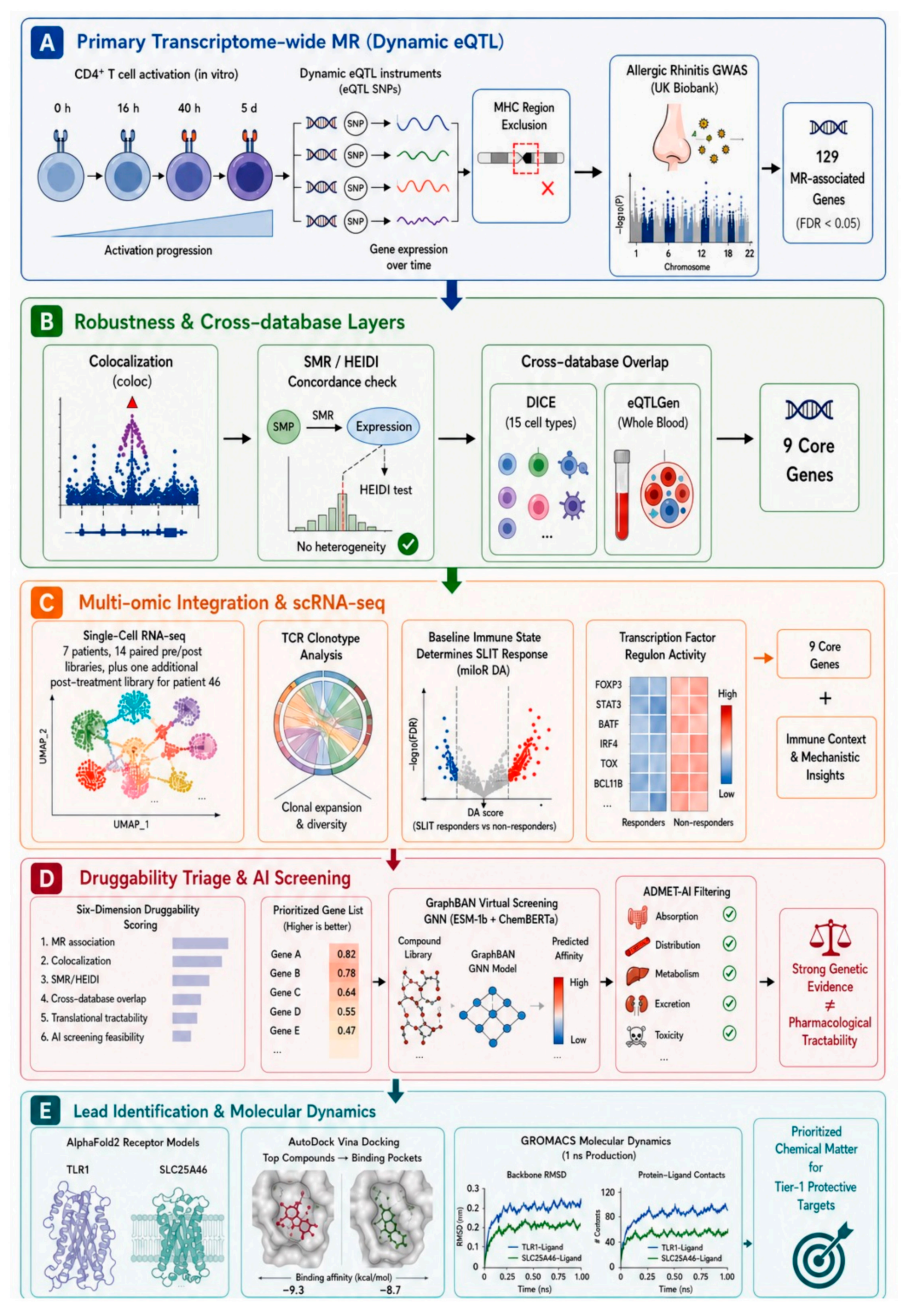
Study overview and analytical framework. Five linked modules summarize the study design and information flow. (**A**) Primary transcriptome-wide Mendelian randomization (MR) using dynamic CD4+ T cell eQTLs across activation states to identify candidate MR-associated genes for allergic rhinitis (AR). (**B**) Robustness and cross-database layers, including colocalization, SMR/HEIDI, cross-database comparison, timepoint analysis, and MHC-region exclusion. (**C**) Single-cell and immune-context contextualization, including scRNA-seq response-state comparison, TCR clonotype structure, and orthogonal neighborhood-level analysis. (**D**) Translational triage, including target ranking and tractability assessment. (**E**) Structure-based inspection of prioritized targets through docking and short molecular dynamics simulations.

Here we used a staged strategy to move from broad association discovery to a shorter list of mechanistically and translationally useful candidates. We first performed transcriptome-wide MR using dynamic CD4+ T cell eQTL and allergic rhinitis GWAS. We then added locus-level assessment by colocalization and SMR/HEIDI, tested cross-database overlap in DICE, the Database of Immune Cell Expression, Expression quantitative trait loci and Epigenomics, and in eQTLGen, a large whole-blood eQTL consortium, examined timepoint-specific effects, screened for metabolite-mediated pathways, placed the prioritized genes in single-cell and paired TCR context, and finally performed restrained computational follow-up on representative protective targets (Figure 1; [15,16]).

## 2. Materials and Methods

### 2.1. Study Design

We used a five-module workflow: primary transcriptome-wide MR, locus-level robustness testing, metabolite mediation screening, single-cell and TCR contextualization, and focused computational follow-up. The workflow was designed to move from broad gene discovery to a smaller set of candidates that could be evaluated for genetic credibility, temporal specificity, cellular context, treatment-response relevance, and tractability. The study follows the STROBE-MR (Strengthening the Reporting of Observational Studies in Epidemiology using Mendelian Randomization) reporting guideline, and the completed checklist is provided as a Supplementary File.

### 2.2. Genetic Instruments and Primary MR

Primary instruments came from the dynamic CD4+ T cell eQTL resource of Soskic and colleagues, which includes 46 expression profiles across 0 h, LA, 16 h, 40 h, and 5 d activation states in 119 donors [12]. Allergic rhinitis summary statistics were taken from UK Biobank Quickdraws (GCST90468131), an outcome setting that builds on earlier large-scale allergic-disease GWAS work in hay fever, asthma, and eczema [9,10]. Variants in the MHC interval on chromosome 6 (25.5–34 Mb, GRCh38) were excluded before downstream MR. Instruments were selected within a ±500 kb cis window at permutation FDR < 0.05 and F statistic ≥ 10, followed by harmonization in the standard TwoSampleMR framework with action 2 [17], in which palindromic variants were resolved by allele frequency where possible and otherwise excluded. Linkage disequilibrium clumping used r^2^ = 0.001 in a 500 kb window against the 1000 Genomes European reference, and Steiger directionality filtering was applied with exposure sample size 119 and outcome sample size 394,626. Single-instrument pairs were analyzed with the Wald ratio, and multi-instrument pairs with IVW, weighted median, weighted mode, MR-Egger, and MR-RAPS. Benjamini–Hochberg (BH) FDR control was applied across all 50,654 gene-profile tests. The outcome GWAS summary statistics were generated from the UK Biobank by the Quickdraws scalable variational-inference pipeline [18]. The GWAS Catalog record (GCST90468131) comprises 394,626 European-ancestry individuals and approximately 13.3 million imputed variants on GRCh38; only the total sample size is reported, and effects are per-allele log odds ratios with standard errors. Because individual case/control counts are not provided, we used an approximate case fraction of 20% (consistent with the UK Biobank self-reported phenotype) as the colocalization case fraction. Summary statistics were downloaded from the GWAS Catalog study page (https://www.ebi.ac.uk/gwas/studies/GCST90468131, accessed on 20 May 2026). The exposure (Soskic et al. [12], 119 European donors) and outcome (UK Biobank) cohorts are independent, with no sample overlap. Exposure effects are expressed per effect allele on the gene-expression scale of the Soskic eQTL resource (tensorQTL slopes), so MR estimates are reported as log odds ratios per unit change in genetically predicted expression, with standard errors; odds ratios with 95% confidence intervals follow directly by exponentiation.

### 2.3. Colocalization, SMR/HEIDI, Cross-Database Comparison, and Timepoint Analysis

Colocalization used coloc under the case–control specification applied throughout the project [19]. SMR and HEIDI followed the summary-data framework introduced for eQTL-trait integration [20]. Cross-database comparison used DICE, which provides eQTL maps for sorted adaptive and innate immune cell subsets, and eQTLGen, which provides large-sample whole-blood cis- and trans-eQTL summary statistics [15,16]. Timepoint heterogeneity across the dynamic eQTL panels was assessed using the project-level summary tables and corresponding heterogeneity statistics.

### 2.4. Metabolite Mediation

Gene-to-metabolite MR was performed for the MR-significant genes against the project metabolite panel of 314 plasma metabolite traits (discovery sample of 8176 European-ancestry individuals; effects reported as standardized metabolite levels per allele) [21]. Significant indirect paths were then tested in a second metabolite-to-AR step, followed by metabolite-level colocalization. Because the final candidate metabolite lacked shared-variant support, this module was interpreted conservatively. The mediation screen was retained because it tests a plausible mechanistic bridge between gene regulation and allergic phenotype, but the interpretive threshold was deliberately set by the shared-variant analysis rather than by indirect-effect significance alone.

### 2.5. Single-Cell and TCR Analysis

GSE200107 was analyzed as a paired pre/post SLIT dataset of memory CD4+ T cells from 7 Japanese cedar pollinosis patients, whereas GSE273975 was used as a descriptive PBMC reference set for broader immune-context reading rather than formal replication [5,22]. Seurat v5 was used for preprocessing and Harmony-based integration of the GSE200107 libraries [23,24]. The single-cell analyses covered cell-state annotation, baseline enrichment, paired positive-cell shifts, the integrated evidence matrix, and miloR neighborhood differential abundance [25]. Paired TCR analyses classified clonotypes by persistence, emergence, contraction, and expansion, and compared repertoire renewal, diversity, and dominant-state transitions between response groups. For miloR, the k-nearest-neighbor graph was built with k = 30 and d = 20 principal components; neighborhoods were sampled with prop = 0.2 (refined sampling); neighborhood distances were computed with d = 20, and differential abundance was tested with a design of ~group at the library level (each library as a replicate unit; SpatialFDR < 0.05); the paired pre/post structure was not modeled in this contrast. Clonotypes were defined by the dataset’s 10× V(D)J clonotype identifiers; shared, pre-only, and post-only clonotypes were defined by their presence in both, only the pre-treatment, or only the post-treatment repertoire of each patient; for shared clonotypes, expansion and contraction were defined as a greater-than-50% relative change in clonotype cell count between time points (otherwise stable); and dominant-state switching was defined as a change in the clonotype’s most frequent annotated cell state between time points.

For baseline enrichment, cells with detectable expression for a given gene were classified as positive and compared between poor and good responders at the cell-type level with Fisher exact testing and ratio-of-observed-to-expected (Ro/e) enrichment metrics. For paired treatment-response analysis, pre/post positive-cell fractions were calculated per patient and compared between response groups at the gene-cell-type level. TCR analyses additionally tracked shared, pre-only, and post-only clonotypes, summarized clonal diversity metrics, and evaluated dominant-state switching among shared clonotypes. These analyses were designed to determine whether the prioritized genes mapped mainly to stable baseline response states, treatment-induced transcriptional remodeling, or clonotype-level repertoire adaptation. Transcription-factor regulon activity scores were estimated with decoupleR using the DoRothea regulon collection and compared between response groups (Table S11).

### 2.6. Computational Follow-Up

The computational follow-up consisted of two sequential layers: target-level translational triage across all 129 MR-significant genes, followed by structure-guided AI-based screening of two representative protective targets. The goal was not to claim lead discovery. Instead, we asked whether highly ranked MR-associated genes could be separated into targets suitable for biologic or pathway interpretation, targets compatible with small-molecule exploration, and targets that remain modality-limited.

At the target-triage stage, each MR-significant gene was evaluated across six dimensions: MR association strength, colocalization support, SMR/HEIDI status, cross-database overlap, translational tractability (target class, structure availability, and pharmacological precedent), and AI screening feasibility. The inferred direction of therapeutic modulation was recorded as a reported property rather than a scored criterion. This step was used to identify targets whose biological relevance was strong enough to justify translational follow-up, while recognizing that genetic support and tractability are not interchangeable properties.

The AI-screening branch focused on TLR1 and SLC25A46. TLR1 was chosen because it represents an immune receptor with a recognizable domain architecture and clear signaling relevance. SLC25A46 was chosen because it is a genetically supported protective target with limited prior small-molecule framing, and therefore provides a stronger test of whether the screening pipeline can generate actionable structural hypotheses for less characterized proteins.

Virtual screening began with 498,910 compounds from the ZINC-Pin1 library, a Pin1-focused subset of the ZINC collection [26]. After deduplication, 249,455 unique SMILES remained. Compounds were then filtered by Lipinski rule-of-five criteria and by removal of pan-assay interference compounds (PAINS)-like chemotypes, yielding 237,326 compounds for model inference. Protein sequences were encoded into ESM-1b embeddings derived from UniProt entries, compound structures were represented by ChemBERTa-derived molecular features, and compound-target pairs were scored with four independent GraphBAN models. Two models were aligned to BioSNAP interaction data and two to KIBA affinity data (inductive mode, trained-model seed 12), and the consensus score for each pair was the mean prediction across the four models; compounds were rank-ordered by this consensus score without an absolute cutoff. The models were trained in this project following the published architecture and protocol, and the final training checkpoints were frozen and used for all scoring.

Compounds retained after GraphBAN ranking were passed through ADMET-AI v2.0.1 filtering. Nine developability-related endpoints were used in this step: Ames mutagenicity, carcinogenicity, clinical toxicity, drug-induced liver injury, and hERG liability (all required below 0.25); oral bioavailability (above 0.5); half-life (above 0 h); plasma protein binding (above 50%); and logP (between 1 and 3). Only compounds that passed all nine criteria were retained for structural follow-up. This reduced the search space to 164 candidate compounds per target and ensured that downstream docking was performed on compounds that were not only predicted binders but also enriched for acceptable early-stage pharmacology.

Structural follow-up was then performed by docking the top ADMET-compliant compounds into target-specific receptor models. For TLR1, two domains of the AlphaFold model AF-Q15399-F1 were considered separately: the extracellular LRR region (residues 27–580) and the intracellular TIR domain (residues 640–786); the per-residue confidence of the model was checked against the database-reported pLDDT values (mean pLDDT 87.0 for the full-length, 92.0 for the LRR domain, and 80.2 for the TIR domain). For SLC25A46, two structural strategies were used: a full-length AlphaFold-based model (AF-Q96AG3-F1) and a focused mitochondrial carrier family domain approach informed by homology. Structure preparation consisted of domain trimming followed by PDBQT conversion with Meeko 0.7.1, and ligands were prepared with RDKit (ETKDGv3 conformers, UFF optimization) and Meeko. Docking was performed with AutoDock Vina 1.2.5 [27] using domain-specific search boxes chosen to reflect plausible ligand-accessible pockets (exhaustiveness = 32, nine output modes, energy range = 4). The docking-box coordinates were derived from reference structures rather than chosen manually: the TLR1 LRR box from the TLR1-TLR2-Pam3CSK4 complex (PDB 2Z7X; center −19.79, −19.38, −31.71 Å; size 23.37 × 20.00 × 20.00 Å) and the TLR1 TIR box centered on the BB-loop signaling surface, cross-checked against PDB 1FYV and pocket detection (center −105.20, 9.70, 77.41 Å; size 22.73 × 20.00 × 20.00 Å). The top-ranked compounds from each docking line were then advanced into explicit-solvent molecular dynamics simulations in GROMACS 2025.4 [28] using the AMBER99SB-ILDN force field with TIP3P water and NaCl neutralizing ions; each complex was energy-minimized and equilibrated (100 ps NVT, 100 ps NPT) before production. Production simulations of 1 ns were performed at 2 fs; the TLR1 TIR complex was simulated in triplicate and the SLC25A46 complex in duplicate, with independent velocity seeds 20260429–20260433, to assess pose-stability reproducibility. These simulations evaluated short-timescale pose stability, protein-ligand contacts, and structural drift relative to the starting docked pose.

These computational analyses were interpreted as prioritization evidence for downstream biochemical and functional testing. The AI-based workflow was used to narrow a large chemical search space, compare candidate targets on tractability grounds, and generate ligand-level hypotheses for follow-up experiments. It was not used to claim validated binding or therapeutic efficacy.

## 3. Results

### 3.1. Transcriptome-Wide MR Identified a Broad Allergic Rhinitis Gene Landscape in Activated CD4+ T Cells

The overall design comprised five connected modules spanning association discovery, robustness testing, metabolite mediation screening, single-cell contextualization, and translational prioritization (Figure 1). Starting from 46 dynamic CD4+ T cell expression profiles, we harmonized the eQTL instruments against allergic rhinitis GWAS summary statistics. We then removed variants in the major histocompatibility complex (MHC) interval, applied linkage disequilibrium (LD) clumping and directionality filtering, and retained 50,654 gene-profile pairs representing 10,328 unique genes for the primary scan.

**Figure 2:**
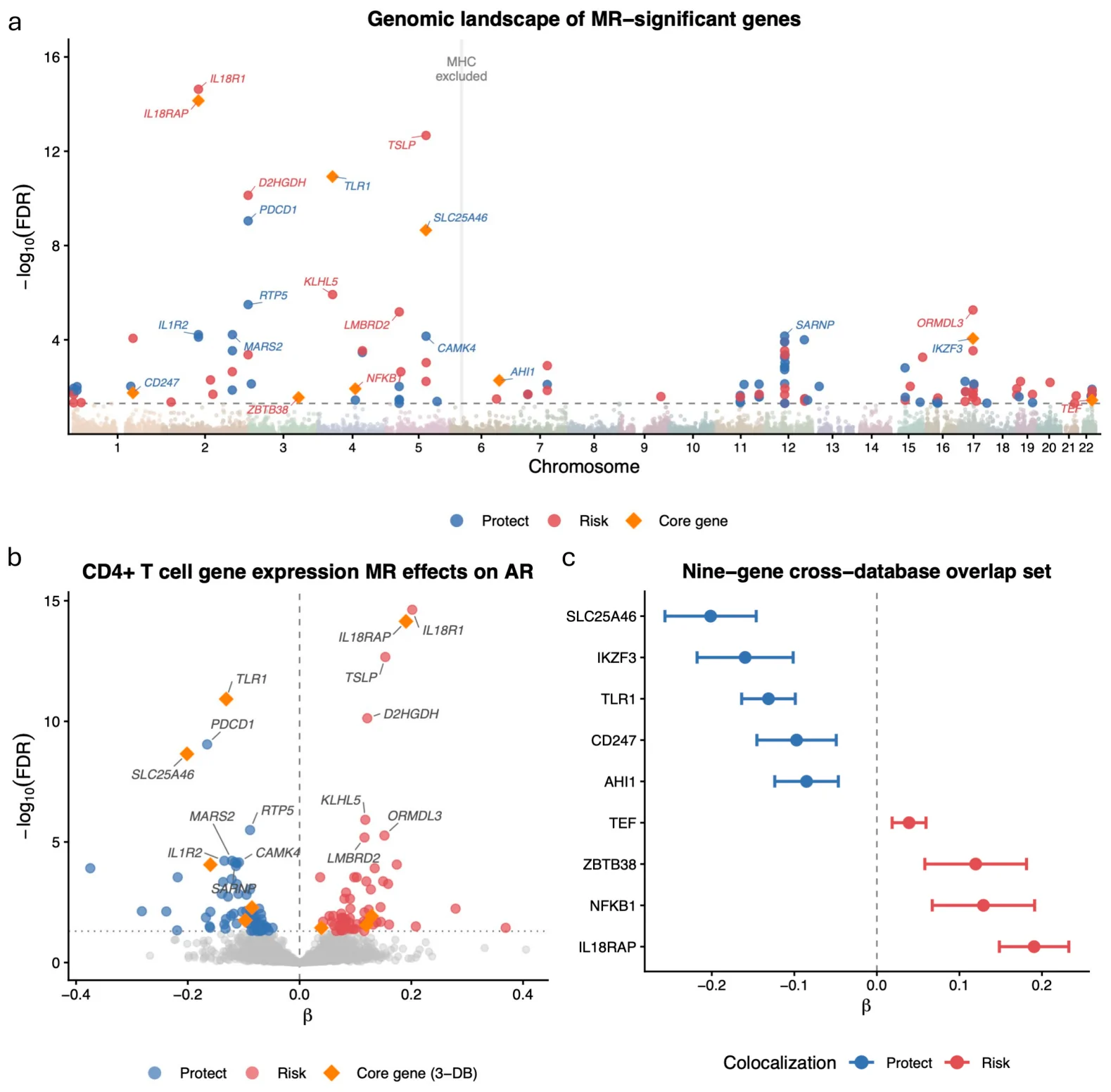
Transcriptome-wide MR identifies 129 CD4+ T cell genes associated with allergic rhinitis. (**a**) Genomic landscape of MR-significant genes across the autosomes after MHC exclusion. Orange diamonds indicate the nine genes later retained in the three-database overlap. (**b**) Volcano plot of the best MR profile per gene among the 129 FDR-significant genes. Blue points indicate protective effects and red points indicate risk-increasing effects. Diamonds indicate the nine core genes retained in the three-database overlap. (**c**) Forest plot of effect estimates for the nine core genes shared across the Soskic, DICE, and eQTLGen analyses.

This screen identified 129 genes associated with allergic rhinitis at FDR < 0.05 (Figure 2). These genes were distributed across the autosomes rather than concentrated in a single residual hotspot, indicating that the signal remained genetically broad after explicit MHC exclusion (Figure 2a). The same genes were also well distributed in effect-size and significance space, with both protective and risk-increasing signals represented (Figure 2b). Risk-side examples included IL18R1, IL18RAP, and TSLP, whereas protective examples included TLR1, PDCD1, and SLC25A46. This spread suggests that the pipeline captures a wider immune-regulatory program rather than a single canonical pathway.

The orange-highlighted subset in Figure 2a marks the genes later retained in the three-database overlap and separates the broad discovery layer from the smaller nine-gene overlap set. The primary MR estimates for those nine genes are summarized in the forest plot in Figure 2c, which provides the bridge between the transcriptome-wide scan and the final overlap-prioritized set.

### 3.2. Colocalization and SMR/HEIDI Reduced the Candidate Set to a More Credible Subset

**Figure 3:**
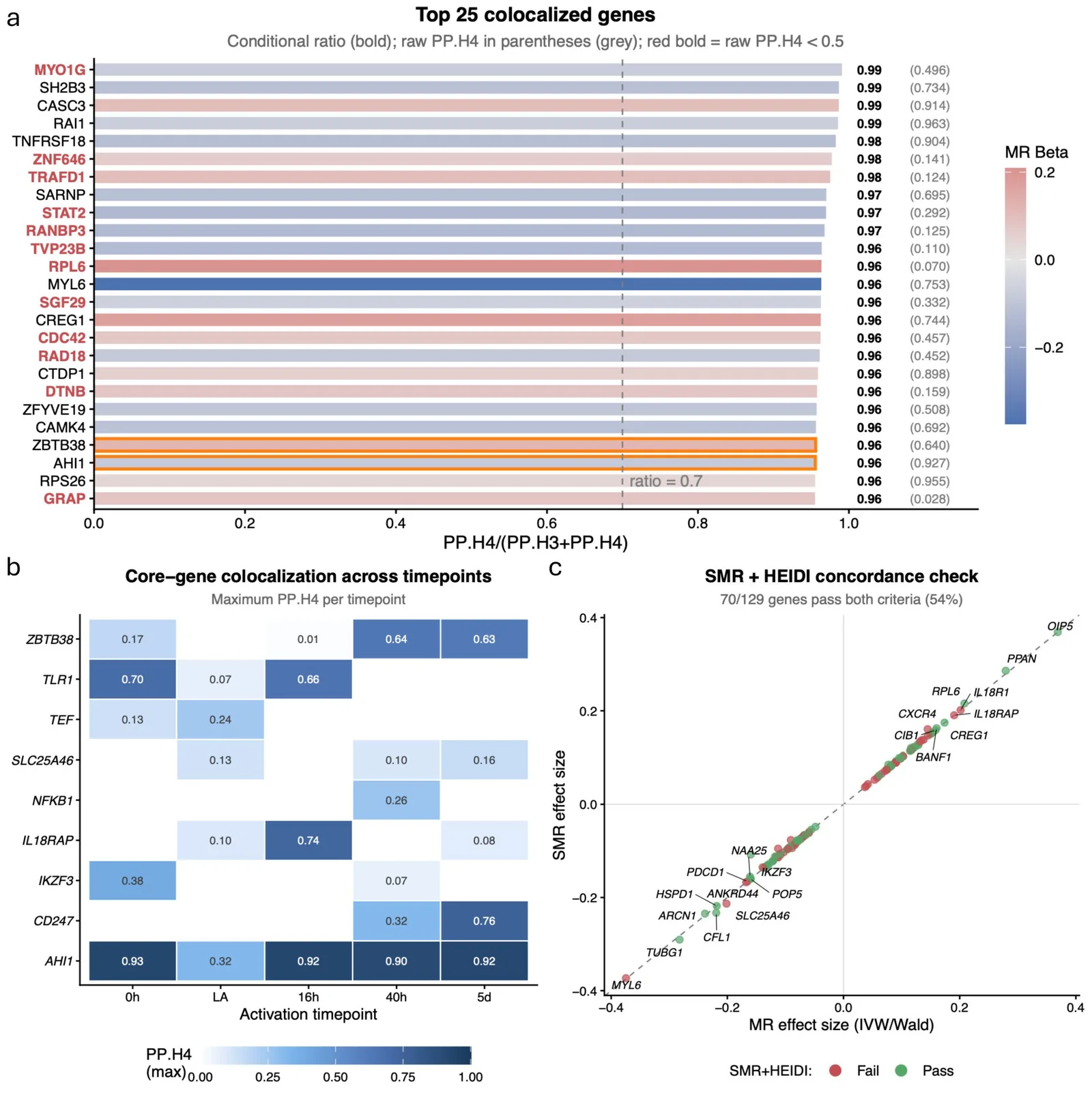
Colocalization and SMR/HEIDI refine the primary MR signal set. (**a**) Top 25 genes ranked by the coloc conditional ratio PP.H4/(PP.H3 + PP.H4) (per-gene maximum across Soskic CD4+ T cell eQTL profiles), restricted to colocalization-significant pairs (*n* = 418 of 944 pairs; conditional ratio > 0.7). Each bar reports the conditional ratio (bold) and the raw PP.H4 (gray). Bars are colored by MR effect direction (blue, protective; red, risk-increasing; IVW, FDR < 0.05). Genes of the core three-database overlap set (*n* = 9) are outlined in goldenrod (here: ZBTB38, AHI1); Genes with low absolute PP.H4 (<0.5) are labeled in bold red. (**b**) Colocalization heatmap across CD4+ T cell activation time points for representative core allergic rhinitis genes. (**c**) Comparison between MR effect size and SMR effect size for MR-significant genes. Green points passed both SMR and HEIDI criteria (70 of 129 genes, 54%); red points failed at least one criterion.

MR significance alone is not sufficient to infer a shared causal variant. We therefore asked which associations remained supported after locus-level assessment. The top 25 genes ranked by colocalization support included recurrent immune loci such as ORMDL3, IL18R1, TSLP, and STAT6 (Figure 3a). The color scale overlays MR direction and magnitude on the colocalization ranking and highlights loci with both strong posterior support and large estimated effects.

Mapping colocalization support across the five canonical activation timepoints added a second layer of resolution (Figure 3b). Several loci showed concentrated support at specific phases of activation rather than flat support across all conditions. This is a practical advantage of the dynamic design, because loci that would appear as single static signals in conventional blood eQTL resources can be resolved into temporally structured effects.

Because the SMR estimate reuses the same top cis-SNP as the single-instrument Wald-ratio MR estimate, it is best interpreted as a concordance check rather than an independent validation layer; the HEIDI statistic contributes the additional diagnostic information by testing heterogeneity across the remaining cis-SNPs. Seventy of the 129 MR-significant genes passed both criteria (p_SMR < 0.05 and p_HEIDI > 0.05; Supplementary Table S5), and the overall correspondence between MR and SMR estimates was strong. The same panel also shows genes that failed this filter and helps separate more credible candidates from associations that may still reflect local LD complexity or weaker instruments. Taken together, these filters convert a transcriptome-wide discovery list into a more interpretable hierarchy (Figure 3).

### 3.3. Cross-Database Overlap Identified a Nine-Gene Set and Revealed Timepoint-Dependent Effects

**Figure 4:**
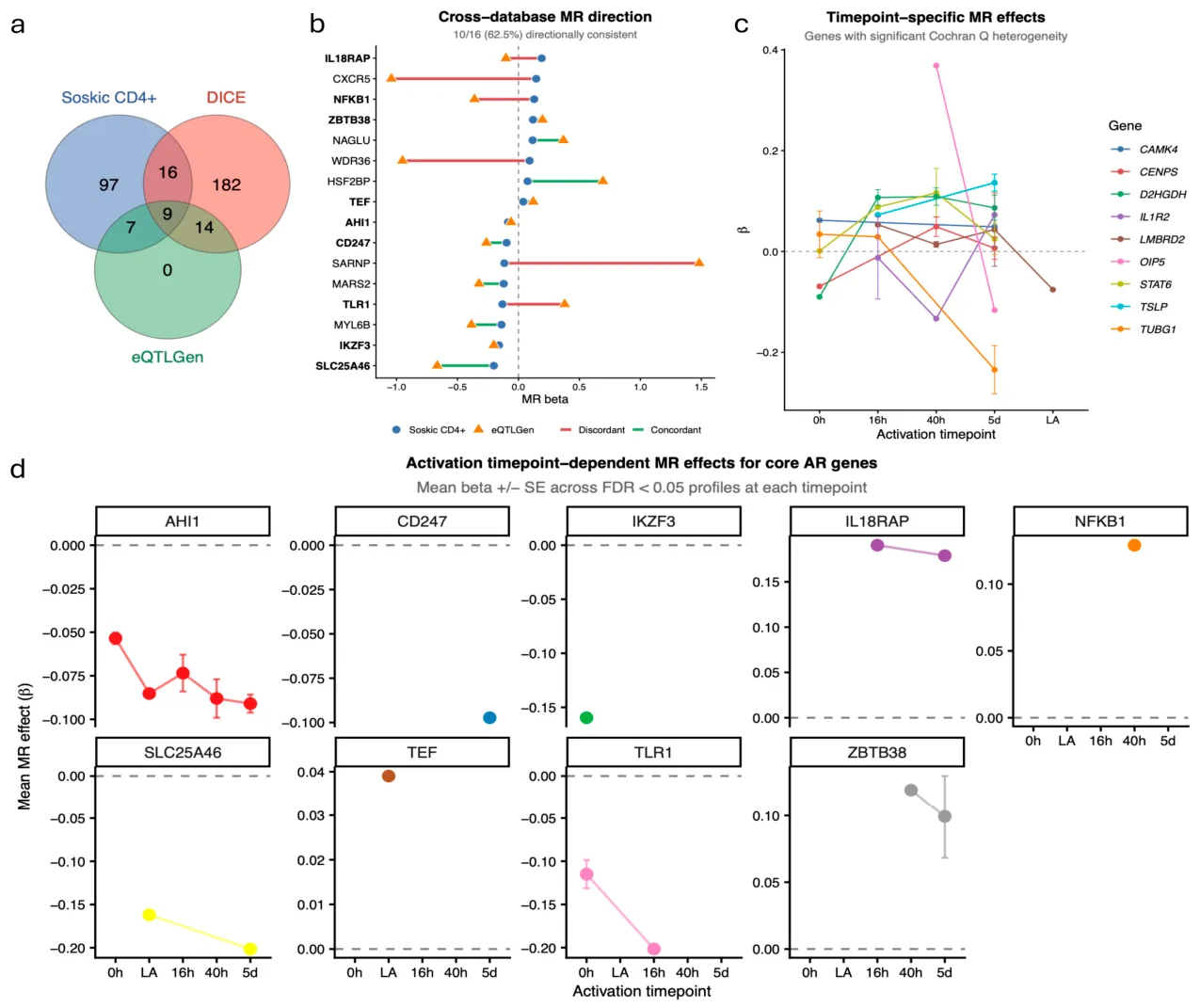
Cross-database overlap and activation-state dependence. (**a**) Venn diagram of MR-significant genes in the Soskic dynamic CD4+ T cell dataset, DICE, and eQTLGen. (**b**) Cross-database comparison of MR direction between the Soskic dataset and eQTLGen; core genes are shown in bold. (**c**) Timepoint-specific MR trajectories for genes with significant heterogeneity across activation states. (**d**) Small-multiple plots of timepoint-dependent MR effects for representative core genes.

We next asked how stable the MR signals were across independent eQTL resources. For this step, we used DICE, which profiles cis-eQTLs across 15 sorted immune cell populations from healthy donors, and eQTLGen, a whole-blood eQTL meta-analysis spanning 31,684 individuals, as complementary external eQTL resources for the Soskic dynamic CD4+ T cell data [15,16]. The three-way overlap yielded an overlap set of nine genes: NFKB1, IL18RAP, AHI1, IKZF3, SLC25A46, TEF, TLR1, ZBTB38, and CD247 (Figure 4a). This overlap set is much smaller than the primary discovery set and remains the most robust candidate subset, although individual genes varied in their support across sensitivity layers: four of the nine genes (IL18RAP, SLC25A46, TEF, and ZBTB38) did not pass the HEIDI filter, and the MR-significant profile of TLR1 did not meet the prespecified colocalization threshold (Table 1; Supplementary Tables S2 and S5).

Cross-database overlap was not simply binary. Among the 16 genes significant in both the Soskic and eQTLGen analyses, 10 (62.5%) showed directionally concordant effects; the six discordant genes included NFKB1, IL18RAP, and TLR1 (Figure 4b). Directional discordance may reflect differences in instrument sets, LD structure, statistical power, cellular composition, or horizontal pleiotropy, and we therefore do not interpret it as direct evidence for effector cell-type localization. For TLR1 and NFKB1, the discordance is consistent with their known expression mainly outside CD4+ T cells, but this interpretation remains contextual rather than definitive.

The timepoint analysis added a second dimension (Figure 4c,d). Several genes varied across 0 h, LA, 16 h, 40 h, and 5 d states, with some effects stronger in early activation and others more prominent later. These findings argue against a purely static model of allergic rhinitis susceptibility in CD4+ T cells and show that dynamic eQTL-based MR can suggest not only which genes matter, but also when they may matter most during activation.

### 3.4. Exploratory Metabolite Screening Identified Indirect-Effect Signals, but Failed the Shared-Variant Test

**Figure 5:**
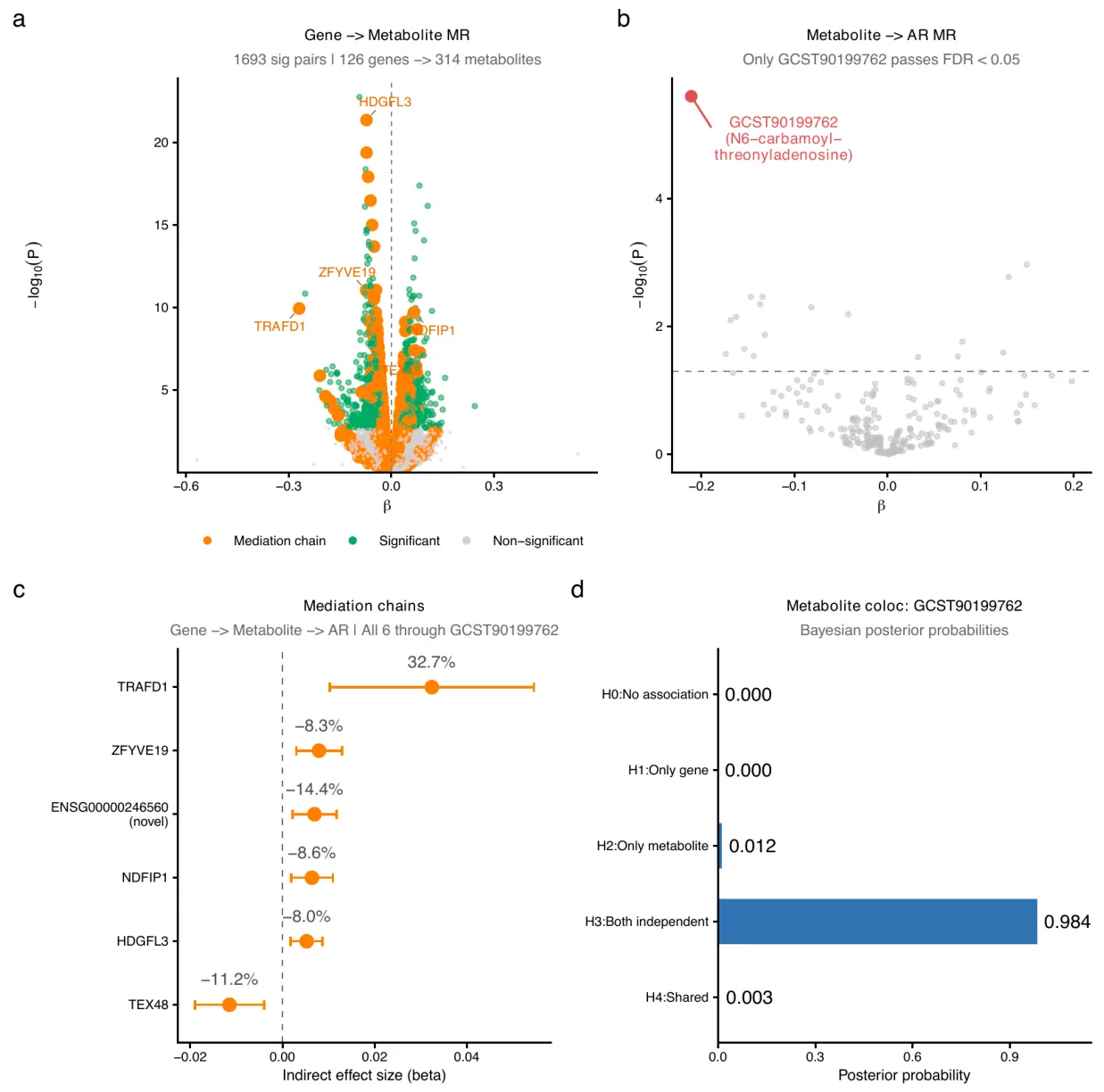
Exploratory metabolite screening identified indirect-effect signals but no shared causal metabolite locus. (**a**) Gene-to-metabolite MR results summarizing 1693 significant pairs. (**b**) Metabolite-to-AR MR results showing the single significant metabolite, the horizontal dashed line indicates the *p* = 0.05 significance threshold (−log_10_(P) = 1.30). (**c**) Indirect effect estimates for 6 gene → metabolite → AR chains. (**d**) Bayesian colocalization posterior probabilities for the candidate metabolite versus the AR GWAS locus.

The metabolite module tested whether part of the gene-to-disease effect might be relayed through circulating metabolic traits [21]. The first screen produced 1693 significant gene-to-metabolite pairs across 126 genes and 314 metabolite GWAS included at this stage (Figure 5a). This signal space was large enough to justify follow-up and suggested that the allergic rhinitis gene set was metabolically connected rather than transcriptionally isolated.

The second filter tested metabolite-to-AR effects, and only one metabolite, N6-carbamoylthreonyladenosine (GCST90199762), remained significant after multiple-testing correction (Figure 5b). The final display showed six surviving gene-to-metabolite-to-AR paths within the mediation framework (Figure 5c). The indirect effects were not uniform in direction or magnitude, which argues against a simple one-metabolite narrative.

The key adjudication came from colocalization of GCST90199762 with the allergic rhinitis locus, where the posterior was dominated by H3 rather than H4, indicating support for distinct causal variants rather than a shared one (Figure 5d). The mediation statistics were therefore worth reporting, but the shared-variant test argued against a stable locus-level mediated mechanism and prevented over-interpretation of the indirect paths.

### 3.5. Single-Cell and Paired TCR Analyses Linked Prioritized Genes to Baseline Response State and Repertoire Remodeling

**Figure 6:**
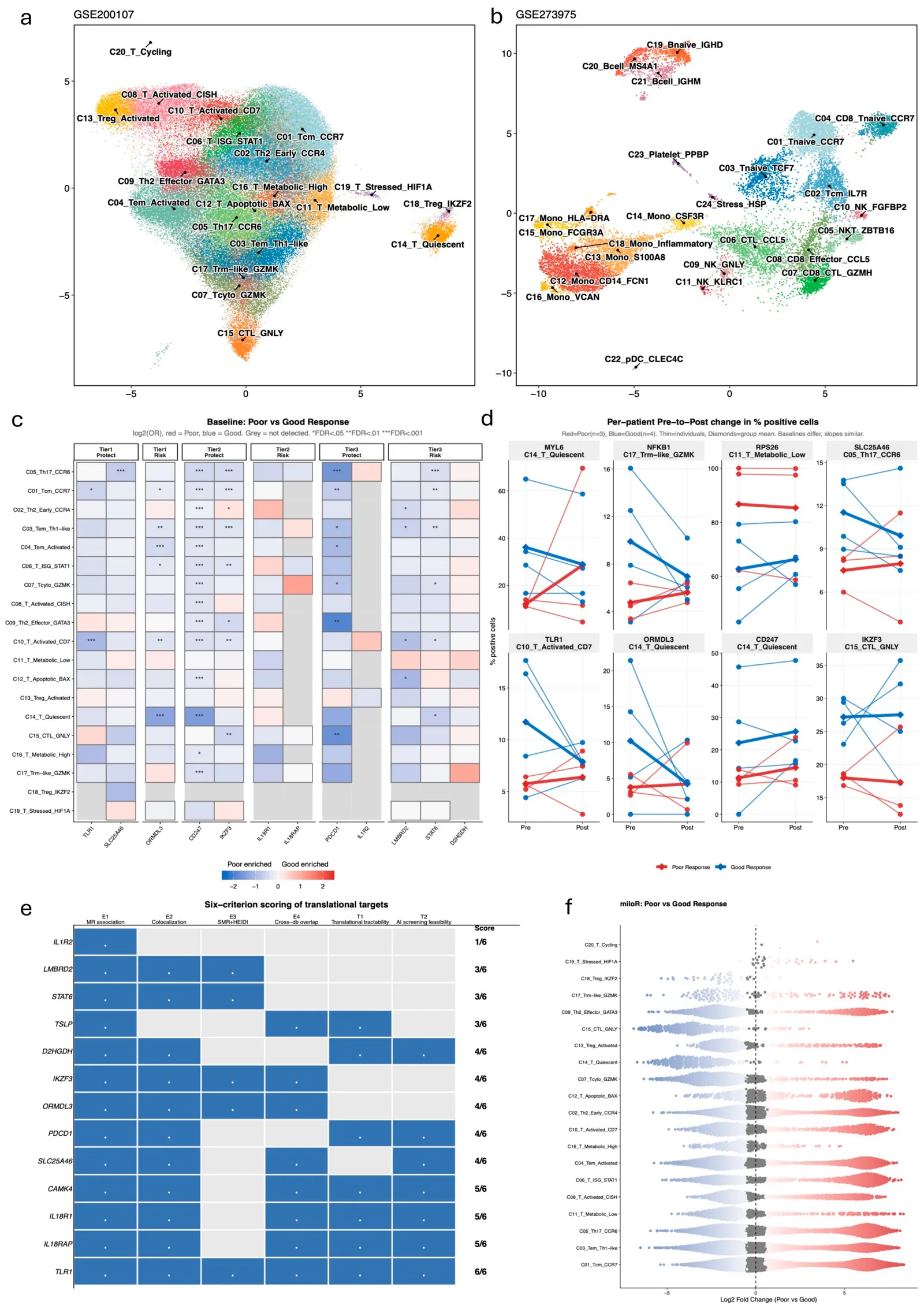
Single-cell contextualization links prioritized MR genes to response-state structure. (**a**) UMAP of GSE200107, comprising 117,771 CD4+ memory T cells annotated into 20 subtypes. (**b**) UMAP of GSE273975, comprising 16,412 PBMCs from two patients (four libraries), used as a small descriptive cross-dataset reference. (**c**) Baseline poor-versus-good enrichment heatmap (log2 OR) for prioritized MR genes; gray tiles, not detected; * FDR < 0.05, ** FDR < 0.01, *** FDR < 0.001. (**d**) Per-patient pre-to-post change in positive-cell percentages for representative gene-cell-type pairs. (**e**) Evidence matrix scoring the 13 candidate genes against six criteria: E1, MR association (FDR < 0.05, IVW); E2, coloc conditional ratio PP.H4/(PP.H3 + PP.H4) > 0.7; E3, SMR+HEIDI (p_SMR < 0.05 and p_HEIDI > 0.05); E4, cross-database overlap (Soskic + eQTLGen); T1, translational tractability; T2, AI screening feasibility. Filled tiles indicate a met criterion. Rows are sorted by total score (x/6), identical to revised Table S13; TLR1 ranks first (6/6) and IL1R2 last (1/6). (**f**) miloR neighborhood differential abundance analysis comparing Poor and Good response groups at baseline.

We next placed the MR-prioritized genes in cellular context using paired single-cell RNA sequencing (scRNA-seq) from GSE200107 and a descriptive peripheral blood mononuclear cell (PBMC) reference dataset, GSE273975. GSE200107 comprised 15 PBMC libraries from 7 Japanese cedar pollinosis patients (14 paired pre/post libraries plus one additional post-treatment library for patient 46), sampled before and after sublingual immunotherapy (SLIT), with paired scRNA-seq and TCR information available for direct response-state analysis [22]. The resulting GSE200107 atlas resolved 117,771 CD4+ memory T cells into 20 annotated subtypes spanning central memory, Th2-like, Th17-like, activated, quiescent, cytotoxic, and regulatory states (Figure 6a). GSE273975 was used as a smaller PBMC reference set to help separate CD4+ T cell-restricted patterns from broader blood-cell expression patterns, but not as a formal inferential replication cohort (Figure 6b). GSE273975 comprises four libraries from two patients treated with Peiyuan Tongqiao Decoction and was used only as a small, unrelated descriptive PBMC reference. A descriptive comparison between the GSE200107 (SLIT) and GSE273975 (TCM decoction) datasets is provided in Supplementary Figure S10.

In a cell-level analysis treating individual cells as observations (561 gene-cell-type tests; 88 unique genes; all 561 cell-level Fisher tests reached nominal significance), several protective genes from the higher-priority tiers appeared depleted in poor responders before treatment (Figure 6c; Supplementary Table S7). For example, cell-level estimates suggested higher CD247-positive fractions in Good responders across 14 subtypes, including C05_Th17_CCR6 (57.3% versus 40.1%) and C01_Tcm_CCR7 (54.2% versus 43.1%); IKZF3 and SLC25A46 showed narrower Good-enriched patterns, and TLR1 remained detectable mainly in activated compartments, most notably C10_T_Activated_CD7 (11.8% versus 5.7%). The risk-side gene RPS26 was broadly enriched in poor responders at the cell level, whereas ORMDL3 showed a discordant pattern. Because cell-level tests treat thousands of correlated cells from the same patient as independent observations, we repeated the baseline comparison at the patient level using per-patient pseudobulk summaries (*n* = 4 good vs. 3 poor responders). No gene-cell-type pair survived FDR correction in the patient-level analysis (minimum FDR = 0.209; two pairs at nominal *p* < 0.05; Supplementary Table S7), reflecting the limited statistical power of the seven-patient design. The baseline cell-level enrichment is therefore presented as a hypothesis-generating observation concerning pretreatment immune configuration rather than as an established responder signature. The broader other-tier cell-level pattern is shown in Supplementary Figure S8.

We next asked whether treatment-induced change differed systematically between response groups (Figure 6d). Across 15,827 paired observations from 7 patients, no gene-cell-type pair survived multiple-testing correction for between-group delta differences (Supplementary Tables S8 and S9). Only 8 pairs reached nominal significance, and these were scattered across genes and cell states without a coherent Tier 1–3 pattern. Within-patient pre-to-post trajectories were present, but group-level slopes were largely parallel. The genetic and cellular evidence was then integrated into the summary matrix in Figure 6e, which summarizes the multi-layer genetic and cellular evidence supporting CD247, IKZF3, SLC25A46, and TLR1 as biologically interpretable candidates. A neighborhood-level differential-abundance analysis using miloR provided complementary compositional information (Figure 6f; Supplementary Table S10). A total of 5384 of 17,789 neighborhoods were differentially abundant between poor and good responders at baseline, with the strongest contribution from C01_Tcm_CCR7 and related memory and activated compartments. Because neighborhood abundance reflects compositional and cell-state changes rather than gene-specific expression, miloR results are reported as complementary to—not independent confirmation of—the gene-level enrichment. By contrast, pre- versus post-treatment comparison did not identify a parallel treatment-wide restructuring signal. Because this between-group delta contrast addresses a different estimand (treatment-induced change) than the baseline good-versus-poor comparison, it is reported as a complementary description and was not used to validate or invalidate the baseline findings.

Because GSE200107 also includes paired TCR information, we next asked whether treatment response was reflected in clonotype dynamics. Six patients had matched pre- and post-treatment TCR repertoires available for direct tracking (patient 53 was excluded because paired repertoire data were not available; Supplementary Table S12). At the patient level, newly emergent post-only clonotypes accounted for a mean of 23.3% of the post-treatment repertoire in the three good responders, compared with 3.5% in the three poor responders; individual good-responder values were heterogeneous and strongly influenced by patient 46 (Supplementary Figure S1). Among shared clonotypes, 7.5% expanded after treatment in good responders versus 1.6% in poor responders (pooled clonotype percentages; Supplementary Figure S2). With only three patients per group, these comparisons are descriptive and hypothesis-generating rather than formally inferential.

Diversity and state-transition analyses supported the same interpretation. In good responders, Shannon diversity remained stable to slightly increased despite greater clonal expansion. In poor responders, expansion was accompanied by lower diversity (Supplementary Table S12). Dominant-state switching was common in both groups, affecting 88.7% of shared clonotypes in good responders and 90.1% in poor responders (Supplementary Figures S3 and S4). The most frequent transitions linked central-memory, early Th2-like, Th17-like, and activated states. Additional views connecting clone structure to MR-gene expression are provided in Supplementary Figures S5–S7. Transcription-factor regulon activity was compared between response groups, and NFKB1 regulon activity across CD4+ T cell subtypes is shown in Supplementary Figure S9. These descriptive results suggest a hypothesis—requiring replication in larger cohorts—that clinical response may be associated more strongly with baseline immune configuration and repertoire plasticity than with a uniform treatment-induced transcriptional program.

### 3.6. Focused Computational Follow-Up Prioritized Representative Binders for SLC25A46 and TLR1

The final module was designed as a translational prioritization layer that linked genetic evidence to tractability rather than stopping at target nomination. We reviewed all 129 MR-significant genes across six practical dimensions. These included target class, availability of experimental or modeled structure, pharmacological precedent, inferred direction of therapeutic modulation from the MR effect, strength of multi-layer genetic support, and feasibility of AI-based small-molecule screening.

**Figure 7:**
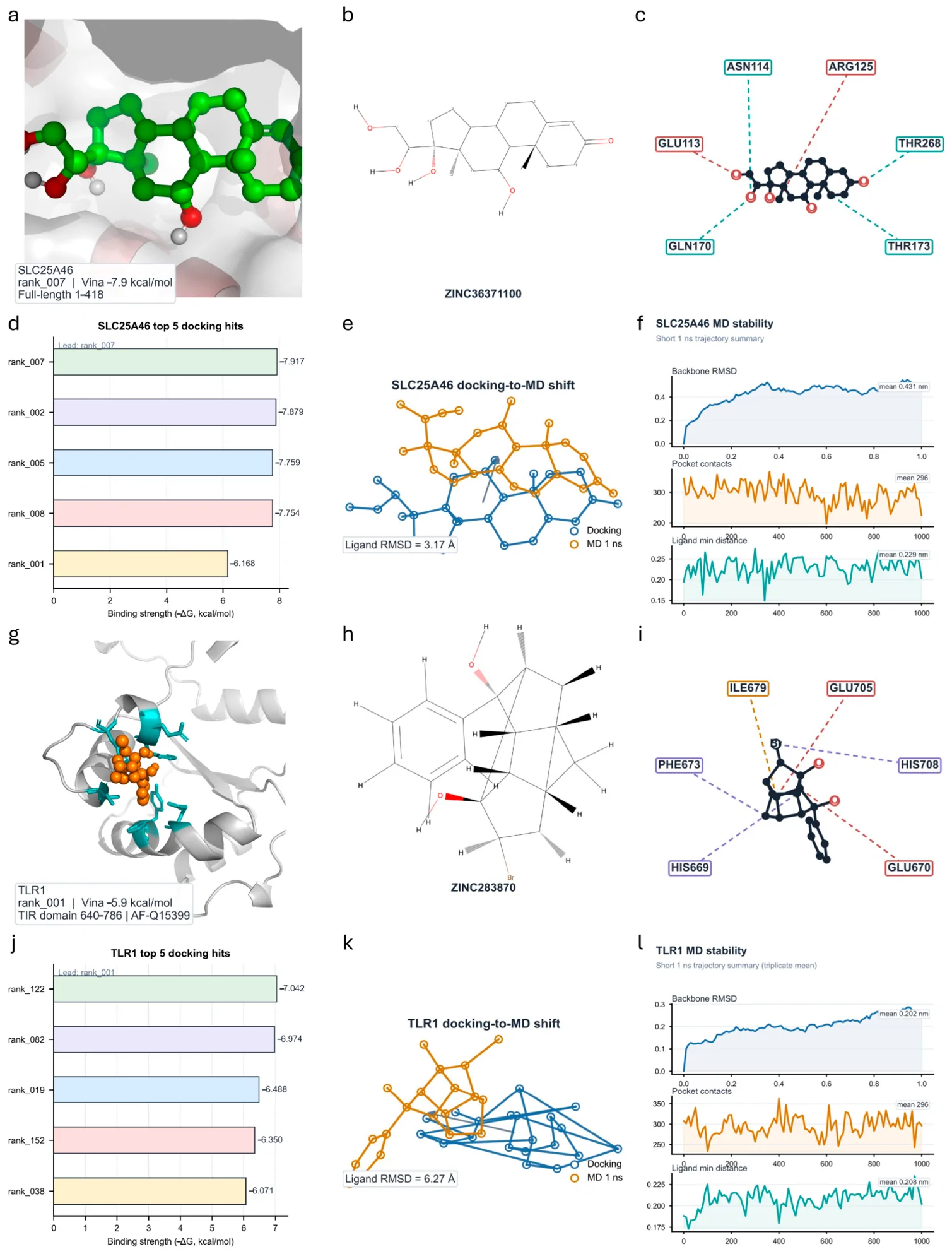
Representative computational follow-up for SLC25A46 and TLR1. (**a**–**f**) SLC25A46: (**a**) 3D binding pose of the prioritized binder candidate, (**b**) 2D structure of ZINC36371100, (**c**) residue-level interaction map, (**d**) top five docked compounds, (**e**) docking-to-MD pose overlay, (**f**) short MD stability traces. (**g**–**l**) TLR1: (**g**) 3D binding pose in the TIR-domain pocket, (**h**) 2D structure of ZINC283870, (**i**) residue-level interaction map, (**j**) top five docked compounds, (**k**) docking-to-MD pose overlay, (**l**) short MD stability traces.

This triage separated several clinically interpretable risk-side genes from the protective-target screening branch. Loci such as TSLP, IL18R1, and STAT6 remained attractive because they align with established inflammatory biology and existing pharmacological precedent. By contrast, the AI-based small-molecule arm focused on protective genes for which classical indication-extension logic was weaker, but where direct structure-guided exploration could still be informative. The target-prioritization framework, including the 13 high-priority genes carried into translational review and the two genes advanced into structure-guided screening, is summarized in Supplementary Table S13. Figure 7 then presents the full screening-to-structure workflow for the two representative case studies, TLR1 and SLC25A46.

The AI-based screening cascade began with 498,910 compounds from the ZINC-Pin1 library. After deduplication, 249,455 unique SMILES remained. Lipinski rule-of-five filtering and removal of pan-assay interference compounds (PAINS)-like chemotypes yielded 237,326 drug-like compounds for model-based scoring. Each compound was encoded with ChemBERTa-derived molecular features [29], and each target protein was encoded with ESM-1b sequence embeddings generated from the corresponding UniProt entry. We then scored compound-target pairs with four independent GraphBAN models [30], including complementary models trained on BioSNAP interaction data and KIBA affinity data. The consensus score for each pair was the mean prediction across the four models. Consensus ranking reduced dependence on any single model or training distribution.

Predicted binders were then filtered through a second AI layer focused on developability rather than affinity alone. ADMET-AI was used to evaluate absorption, distribution, metabolism, excretion, and toxicity (ADMET)-related endpoints [31], including mutagenicity, carcinogenicity, clinical toxicity, liver injury risk, human ether-a-go-go-related gene (hERG) liability, oral bioavailability, half-life, plasma protein binding, and logP. After this step, 164 compounds per target passed all nine filters. This reduction was a practical outcome of the screening module. It compressed a large chemical search space into a smaller, pharmacology-aware subset for downstream docking and molecular dynamics (MD) analysis. The selected leads and their surrounding top-ranked docked compounds are shown in the ranked summary panels of Figure 7d,j, with the full lead-compound summary listed in Supplementary Table S13.

For SLC25A46, we evaluated both a full-length AlphaFold-based pocket [32] and a focused mitochondrial carrier family domain strategy, then advanced the top compounds into docking and short explicit-solvent MD analysis. Figure 7a shows the three-dimensional binding pose of the selected lead in the full-length pocket, and Figure 7b identifies that prioritized candidate as ZINC36371100. The residue-level interaction map in Figure 7c shows the contacts that support the docked pose, whereas Figure 7d places the lead in the context of the top five ADMET-compliant docked compounds. Figure 7e then compares the initial docking pose with the 1 ns MD endpoint, and Figure 7f summarizes the short-timescale stability traces.

For TLR1, the same pipeline pointed to a different translational logic. Docking to the Toll/interleukin-1 receptor (TIR) domain outperformed docking to the extracellular leucine-rich repeat (LRR) region, suggesting that the intracellular signaling domain may offer the more coherent small-molecule entry point in this framework. The retained binder candidate ZINC283870 docked into the TIR-domain pocket at −5.856 kcal/mol and into the LRR region at −4.147 kcal/mol, and triplicate TIR-domain simulations gave a mean backbone RMSD of 0.202 nm with a mean of 296 protein-ligand contacts per frame. Figure 7g shows the selected TIR-domain binding pose, and Figure 7h identifies the corresponding prioritized binder candidate as ZINC283870. The contact map in Figure 7i and the ranked docking summary in Figure 7j show how this lead compares with the other top ADMET-compliant candidates for the same pocket. Figure 7k shows the docking-to-MD pose overlay, and Figure 7l summarizes the corresponding short MD stability traces. We therefore interpret the final compounds as computationally prioritized binder candidates for biochemical follow-up, rather than as pharmacological leads.

## 4. Discussion

This study presents a genetically grounded target-prioritization framework for allergic rhinitis that moves from transcriptome-wide association discovery to a shorter list of candidates, each characterized by a defined evidence profile rather than a single summary claim. The primary output is a list of 129 MR-associated genes identified from 50,654 gene-profile tests across 46 dynamic CD4+ T cell activation profiles. These genes were then filtered through colocalization, SMR/HEIDI, cross-database comparison, and a prespecified six-criterion scoring rubric into a nine-gene cross-database overlap set and, ultimately, two representative structural case studies. The approach is aligned with recent dynamic-CD4 eQTL MR studies in cardiometabolic disease and colorectal cancer [13,14] and extends the framework into allergic rhinitis and into an immunotherapy-response setting in which paired single-cell and TCR data provide biological context for the prioritized genes.

Several features support the framework. First, the exposure captures dynamic, activation-state-specific CD4+ T cell regulation rather than static bulk-blood expression, allowing time-dependent and subset-dependent structure to be resolved. Second, each downstream layer is reported at its true evidential strength. Colocalization is reported with both the raw and conditional posterior probabilities and was subjected to prior-sensitivity analyses; SMR is presented as a concordance check with HEIDI as the accompanying heterogeneity diagnostic; and cross-database comparison distinguishes significance overlap from directionally concordant overlap (62.5% for the Soskic-eQTLGen intersection). Third, the scoring rubric prespecifies its six criteria and weights, separates genetic evidence from translational tractability, and was tested under alternative weightings, under which the top candidate remained stable. Target-disease pairs supported by human genetics remain more likely to translate than targets nominated without such support [33], but the present design is explicitly prioritization rather than validation.

The biology of the higher-priority genes is coherent with current understanding of allergic disease. TSLP, STAT6, and ORMDL3 sit close to established type 2 inflammatory and allergy-risk pathways, including the canonical 17q21 locus, and the TSLP axis already has clear pharmacological precedent in severe asthma [10,11,34,35]. By contrast, CD247, IKZF3, TLR1, and SLC25A46 point to a more mixed landscape spanning antigen-receptor signaling, lymphocyte differentiation, innate sensing, and mitochondrial regulation. This broader pattern fits the view that allergic rhinitis is not driven by a single effector axis, but by interactions among epithelial, innate, and adaptive programs [1,6,7]. Within the overlap set, evidence strength varies across genes: five of the nine genes passed SMR and HEIDI, and the MR-significant profile of TLR1 did not meet the colocalization threshold, so individual genes should be read with their full evidence profile rather than as uniformly supported.

The single-cell module provides exploratory context rather than independent validation. In a cell-level analysis, baseline good-versus-poor differences appeared broad and were consistent with a response phenotype partly encoded before treatment. However, a patient-level reanalysis (four good versus three poor responders) found no gene-cell-type pair surviving multiple-testing correction, and we therefore regard the baseline pattern as hypothesis-generating only. This experience also illustrates the general risk of treating thousands of correlated cells as independent observations. The paired TCR data, described at the patient level for six patients with available repertoires, similarly suggested repertoire renewal and remodeling in good responders but remain descriptive with three patients per group. Together, these observations are compatible with prior work showing that successful allergen immunotherapy is accompanied by immune-state reprogramming [4,22,36], but they require confirmation in larger independent SLIT-response cohorts.

The computational module adds a separate tractability layer. Genetic studies often stop at target nomination, or move too quickly from biological plausibility to therapeutic speculation. Here, model-based ranking, ADMET-aware filtering, structural docking, and short MD simulations were used to distinguish biologically interesting targets from those that could support chemically actionable hypotheses. The contrast among prioritized genes illustrates the point: CD247 and IKZF3 were compelling on the genetic and cellular axes yet remain difficult from a classical small-molecule perspective, whereas TLR1 and SLC25A46 offered sufficient structural definition to support screening and pose-based refinement. Strong MR-based genetic support therefore does not guarantee equal druggability.

The study also provides useful negative information. The metabolite mediation module initially appeared promising, but the lack of colocalization support, the absence of a shared causal variant, and the predominance of negative mediated proportions prevented over-interpretation of a shared metabolite mechanism. Likewise, the computational screening module prioritized binder candidates but did not justify claims of validated pharmacological action. These boundaries preserve the credibility of the positive findings and clarify where the evidence remains hypothesis-generating rather than decisive. The present work is therefore best viewed as a prioritization study with a translational orientation, not as a lead-discovery or mechanism-proving study.

Several limitations warrant emphasis. The primary MR estimates rest predominantly on single-SNP instruments, which precludes standard multi-instrument sensitivity analyses for horizontal pleiotropy; Steiger filtering, HEIDI, and colocalization therefore carry the robustness burden for these pairs. Colocalization support is sensitive to the prior on shared causal variants, with the number of supported pairs falling from 418 at the default prior to 20 under a stricter prior, and many supported pairs have low absolute PP.H4 values; these pairs are flagged in the Supplementary Tables and should be interpreted cautiously. Our HEIDI implementation follows the original formulation but does not apply LD pruning, and the activation-profile estimates share donors and outcome GWAS data, so the Cochran Q statistics are descriptive rather than calibrated tests. Cross-database overlap showed directional concordance in only 62.5% of the Soskic-eQTLGen intersection. The single-cell analyses rest on seven patients; the baseline enrichment did not survive patient-level correction; no independent SLIT-response cohort was available; and the TCR comparisons include only three patients per group. The computational compounds are unvalidated in silico candidates; the MD simulations are short (1 ns production runs), no membrane environment was modeled for SLC25A46, and no docking controls were included. Finally, all genetic analyses used European-ancestry resources, and generalizability to other populations requires verification.

Within these boundaries, the study delivers a transparent and reproducible prioritization: among the 129 MR-associated genes, IL18R1 and IL18RAP represent the strongest risk-side receptor candidates, TSLP combines moderate genetic support with exceptional pharmacological precedent, and TLR1, SLC25A46, CD247, and IKZF3 form the most consistently supported protective-side candidates. Each gene is now reported with its complete evidence profile, so that downstream experimental programs can invest in functional validation where the genetic and tractability evidence is strongest.

## Figures and Tables

**Table 1:** Nine-gene cross-database overlap set: evidence summary across MR, colocalization, and SMR/HEIDI sensitivity analyses. * For TLR1, the MR-significant profile (CD4_Memory_uns_0h) did not meet the prespecified colocalization threshold (conditional ratio 0.518); colocalization support was observed for other activation profiles (TN_0h: 0.867; CD4_Naive_stim_16h: 0.870; Supplementary Table S2).

Gene	Ensembl ID	Best Activation Profile	MR Direction	Soskic FDR	DICE FDR (Cell Type)	eQTLGen FDR	Coloc Ratio (Raw PP.H4)	SMR *p*	HEIDI *p*	SMR + HEIDI Pass
CD247	ENSG00000198821	CD4_Naive_stim_5d	Protective	0.018	1.87 × 10^−3^ (TREG_NAIVE)	7.29 × 10^−5^	0.951 (0.762)	3.37 × 10^−3^	0.934	Pass
IKZF3	ENSG00000161405	CD4_Memory_uns_0h	Protective	8.77 × 10^−5^	1.2 × 10^−3^ (TREG_NAIVE)	6.38 × 10^−6^	0.837 (0.383)	3.53 × 10^−3^	1.000	Pass
IL18RAP	ENSG00000115607	TN_16h	Risk	7.14 × 10^−15^	1.25 × 10^−15^ (TH17)	9.25 × 10^−11^	0.905 (0.736)	3.84 × 10^−4^	5.89 × 10^−86^	Fail
NFKB1	ENSG00000109320	TM_ER-stress_40h	Risk	0.012	3.45 × 10^−3^ (M2)	4.28 × 10^−4^	0.736 (0.265)	6.57 × 10^−3^	0.917	Pass
AHI1	ENSG00000135541	TN_IFN_LA	Protective	5.26 × 10^−3^	5.45 × 10^−3^ (B_CELL_NAIVE)	0.012	0.837 (0.321)	6.4 × 10^−3^	1.000	Pass
SLC25A46	ENSG00000164209	TN_cycling_5d	Protective	2.23 × 10^−9^	4.5 × 10^−6^ (MONOCYTES)	8.94 × 10^−6^	0.717 (0.155)	2.24 × 10^−3^	6.31 × 10^−105^	Fail
TEF	ENSG00000167074	TEM_LA	Risk	0.036	0.028 (NK)	0.045	0.735 (0.244)	4.9 × 10^−3^	2.43 × 10^−4^	Fail
TLR1	ENSG00000174125	CD4_Memory_uns_0h	Protective	1.19 × 10^−11^	5.93 × 10^−14^ (TREG_MEM)	4.18 × 10^−14^	0.518 (0.382) *	4.35 × 10^−5^	0.998	Pass
ZBTB38	ENSG00000177311	CD4_Naive_stim_40h	Risk	0.028	0.027 (M2)	0.044	0.956 (0.640)	2.66 × 10^−3^	0.039	Fail

## Data Availability

Primary public resources reused in this study are available from the following repositories: allergic rhinitis GWAS GCST90468131 from the GWAS Catalog (https://www.ebi.ac.uk/gwas/studies/GCST90468131, accessed on 20 May 2026); dynamic CD4+ T cell eQTL from Soskic et al. under EGA accession EGAS00001005827 (https://ega-archive.org/studies/EGAS00001005827, accessed on 20 May 2026); DICE (https://dice-database.org, accessed on 20 May 2026); eQTLGen (https://www.eqtlgen.org, accessed on 20 May 2026); metabolite GWAS from IEU OpenGWAS (https://gwas.mrcieu.ac.uk, accessed on 20 May 2026); and GEO datasets GSE200107 and GSE273975. Analysis scripts are deposited on GitHub: https://github.com/dr-gu/CD4-MR-AR-public (accessed on 20 May 2026). Derived summary tables and figures underlying the main and supplementary analyses are included in the submission package.

## Supplementary Materials

The following supporting information can be downloaded at: https://www.mdpi.com/article/10.3390/biomedicines14092077/s1, Figure S1: Pre-to-post clonotype persistence, loss, and emergence alluvial summary; Figure S2: Clone-size change among shared clonotypes; Figure S3: Dominant-state transitions of shared clonotypes in good responders; Figure S4: Dominant-state transitions of shared clonotypes in poor responders; Figure S5: MR core-gene expression across clonotype expansion groups; Figure S6: Distribution of representative MR core genes across clone expansion states; Figure S7: Cell-state composition of newly emergent post-treatment clonotypes; Figure S8: Baseline enrichment of top other-tier MR genes; Figure S9: NFKB1 regulon activity across CD4+ T cell subtypes; Figure S10: Descriptive comparison between the GSE200107 (SLIT) and GSE273975 (TCM decoction) datasets. Table S1: MR-significant genes (129 genes); Table S2: Colocalization-supported gene-profile pairs; Table S3: Significant mediation chains; Table S4: Timepoint heterogeneity; Table S5: SMR and HEIDI results; Table S6: CellChatDB reference annotations; Table S7: Baseline Poor-versus-Good enrichment; Table S8: Per-patient paired delta; Table S9: Between-group delta Wilcoxon; Table S10: miloR neighborhood differential abundance; Table S11: TF regulon comparison; Table S12: TCR clonotype dynamics; Table S13: Translational prioritization and AI screening summary. Checklist S1: STROBE-MR checklist.
