# Supplementary material for: Dynamic CD4+ T Cell Genetics Identifies Candidate Therapeutic Targets for Allergic Rhinitis: Mendelian Randomization with Exploratory Single-Cell Contextualization
Source: Biomedicines. 2026 Sep 15;14(9):2077. doi: 10.3390/biomedicines14092077 (PMC13604737; doi:10.3390/biomedicines14092077)
Supplement: Supplementary file 1 [file biomedicines-14-02077-s001.zip › biomedicines-4482591-supplementary/STROBE_MR_checklist S1.pdf]

STROBE-MR Checklist S1

Manuscript: Dynamic CD4+ T cell genetics identifies candidate therapeutic targets for allergic rhinitis: Mendelian randomization with exploratory single-cell contextualization

Note: Completed following the STROBE-MR items (Skrivankova et al., JAMA 2021). Locations are given as section and page; page references follow the manuscript layout, and the section numbers (Sec. X.Y) remain authoritative.

Item	Checklist item	Location in manuscript and content
**Title and abstract**		
1a	Title mentions Mendelian randomization	Title (p. 1): "...Mendelian randomization with exploratory single-cell contextualization"
1b	Abstract summarizes background/objectives/design/data/main results/conclusions	Abstract (p. 1, structured as Background/Methods/Results/Conclusions)
**Introduction**		
2	Scientific background and rationale	Sec. 1 Introduction, first paragraph (p. 2, AR disease burden + the gap for CD4+ T cell dynamic eQTLs)
3	Clear objectives (exposure, outcome, parameters)	Sec. 1, last paragraph (p. 2, staged strategy) + Sec. 4.1 (p. 17, 129 MR-associated candidate genes → nine-gene cross-database overlap set → candidate therapeutic target prioritization)
**Methods: Study design and data sources**		
4	Key elements of study design	Sec. 4.1 (p. 17, five modules: primary MR, robustness, cross-database replication, mediation, single-cell + computational follow-up); Fig 1 (p. 3)
5	Setting, locations, and data time points	Sec. 4.1/4.2 (pp. 17–18, Soskic eQTL five time points 0h/LA/16h/40h/5d; GWAS GCST90468131 and access link); Sec. 4.5 (pp. 18–19, scRNA datasets and sampling time points)
**Participants**		

Item	Checklist item	Location in manuscript and content
6a	Eligibility criteria and sources of outcome/exposure populations	Sec. 4.2 (p. 18, Soskic CD4+ eQTL 46 profiles, 119 donors; GWAS GCST90468131, N = 394,626 European ancestry, UK Biobank, approximate case fraction 20%)
6b	Comparison of source populations (exposure/outcome from independent samples)	Sec. 4.2 (p. 18, two-sample MR; exposure and outcome from independent populations: Soskic 119 donors vs UKB 394,626)
6c	Handling of missing data	Sec. 4.2 (p. 18, GWAS summary statistics report only the total sample size; no missing-data handling steps for summary data)
6d	Sensitivity to selection/attrition (if any)	Not applicable (GWAS/eQTL summary data)
**Genetic variants (instrumental variables)**		
7a	Instrument selection criteria	Sec. 4.2 (p. 18, ±500 kb cis window, permutation FDR < 0.05, F ≥ 10, MHC chr6:25.5–34 Mb exclusion, TwoSampleMR harmonization) + Sec. 2.1 (p. 4, LD clumping + directionality filtering); per-instrument details (F statistic column) in Table S1 and the analysis scripts (GitHub)
7b	Allele harmonization and LD handling	Sec. 4.2 (p. 18, TwoSampleMR action = 2: palindromic variants inferred from EAF, non-inferable excluded; clumping r^2 = 0.001/500 kb/1000 Genomes European panel); per-SNP details in Table S1 (Harmonization status column) and the GitHub scripts
7c	Instrument strength and statistical power	Sec. 4.2 (p. 18, weak-instrument filtering at F ≥ 10; single-SNP Wald ratio in most cases); per-instrument F statistics in Table S1; single-SNP limitation stated in the Discussion limitations paragraph (p. 17)

Item	Checklist item	Location in manuscript and content
7d	Assessment of pleiotropy	Sec. 4.2/4.3 (p. 18, IVW/weighted median/weighted mode/MR-Egger/MR-RAPS/coloc/HEIDI) + Discussion limitations paragraph (p. 17, Steiger filtering; statement that pleiotropy assessment is limited with single-SNP instruments)
**Assumptions**		
8	Statement and assessment of the three core IV assumptions	Sec. 4.1/4.2 (pp. 17–18) + Discussion limitations paragraph (p. 17, relevance = F ≥ 10 filtering (Sec. 4.2) + per-instrument F statistics (Table S1); exclusion restriction = Steiger directionality filtering + HEIDI; independence = discussion of horizontal pleiotropy and multi-method sensitivity analyses)
**Statistical methods: main analysis**		
9a	Main MR methods	Sec. 4.2 (p. 18, Wald ratio/IVW/weighted median/weighted mode/MR-Egger/MR-RAPS, BH FDR across all 50,654 tests) + Sec. 2.1 (p. 4, 50,654 gene-profile tests, FDR < 0.05)
9b	Scale and units of effect estimates	Sec. 4.2 (p. 18, outcome as per-allele log OR; exposure as eQTL effect per allele)
9c	Pleiotropy tests	Sec. 4.2 (p. 18, multi-SNP IVW, weighted median, weighted mode, MR-Egger, MR-RAPS) + Sec. 4.3 (p. 18, HEIDI heterogeneity diagnostic)
9d	Handling of missing data/collinearity	Sec. 4.2 (p. 18) + Sec. 2.1 (p. 4, LD clumping to handle collinearity; no missing data in summary statistics)
**Statistical methods: additional and sensitivity analyses**		
10	Sensitivity analyses	Sec. 4.3 (p. 18, dual coloc criteria, prior sensitivity,

Item	Checklist item	Location in manuscript and content
		SMR+HEIDI, Steiger); Discussion (p. 16 prior-sensitivity statement; p. 17 supporting pairs 418 → 20); Sec. 4.5 (pp. 18–19, patient-level pseudobulk reanalysis; results in Sec. 2.5 p. 12)
11	Other analyses (replication/subgroup/mediation)	Sec. 4.3 (p. 18, DICE/eQTLGen cross-database replication, time-point heterogeneity statistics); Sec. 4.4 (p. 18, metabolite mediation → negative exploration); Sec. 4.5–4.6 (pp. 18–20, scRNA/TCR/computational screening)
12	Software and pre-registration	Sec. 4.1–4.6 (pp. 17–20, TwoSampleMR, coloc, Seurat v5, Harmony, miloR, GraphBAN, ADMET-AI v2.0.1, AutoDock Vina, GROMACS 2025.4); complete scripts and versions at GitHub (dr-gu/CD4-MR-AR-public); the analyses were not pre-registered (stated as such)
13	Ethics approval and informed consent	Institutional Review Board Statement (p. 21, publicly available summary data, no approval required)
**Results**		
14	Descriptive data (numbers of variants/exposure/outcome)	Abstract (p. 1) + Sec. 2.1–2.3 (pp. 4–8, 50,654 gene-profile tests, 129 FDR-significant genes, 418 colocalizing pairs, 70 genes with SMR+HEIDI, nine-gene cross-database overlap set); Table S1 with the complete 50,654 rows
15	Main results	Sec. 2.1–2.3 (pp. 4–8, β, SE, FDR; Fig 2 pp. 4–5; Table 1 p. 20)
16	Other analyses	Sec. 2.4–2.6 (pp. 8–14, negative metabolite results, non-significant patient-level scRNA reanalysis, descriptive TCR results, computational screening)

Item	Checklist item	Location in manuscript and content
17	Sensitivity results	Sec. 2.2 (pp. 5–6, 418 coloc pairs, 70 genes with SMR+HEIDI, HEIDI failure in 4/9 core genes) + Sec. 2.3 (pp. 7–8, directionality concordance 62.5%) + Discussion limitations paragraph (p. 17, prior sensitivity 418 → 20)
**Discussion**		
18	Limitations and their impact on results	Discussion limitations paragraph (p. 17, single-SNP instruments, limited pleiotropy assessment, low absolute PP.H4, HEIDI without LD pruning, descriptive Q tests, single-cell results from 7 patients with non-significant patient-level correction, no independent SLIT cohort, 3 patients per TCR group, unvalidated computational hits, 1 ns MD, no membrane environment for SLC25A46, no docking controls)
19	Interpretation (considering assumption violations)	Discussion (pp. 15–17, genetic support ≠ druggability: p. 17 "genetic support therefore does not guarantee equal druggability"; hypothesis-generating conclusions); Sec. 4.6 (p. 19, genetic evidence and druggability are not interchangeable)
20	Generalizability	Discussion limitations paragraph (p. 17, European-ancestry GWAS/eQTL, "generalizability to other populations requires verification")
**Other information**		
21	Funding	Funding (p. 21, 2022-PUMCH-B-094)
22	Data and code availability	Data Availability Statement (p. 22, GWAS Catalog, EGA EGAS00001005827, DICE, eQTLGen, OpenGWAS, GEO; analysis scripts at GitHub: https://github.com/dr-gu/CD4-MR-AR-public)

Item	Checklist item	Location in manuscript and content
23	Conflicts of interest	Conflicts of Interest (p. 22, none)
