# Supplementary material for: Dynamic CD4+ T Cell Genetics Identifies Candidate Therapeutic Targets for Allergic Rhinitis: Mendelian Randomization with Exploratory Single-Cell Contextualization
Source: Biomedicines. 2026 Sep 15;14(9):2077. doi: 10.3390/biomedicines14092077 (PMC13604737; doi:10.3390/biomedicines14092077)

Supplementary Figures

Figure S1. Pre-to-post clonotype persistence, loss, and emergence alluvial summary. Axis order corrected so that Pre precedes Post. Good: 920 lost / 5,170 new; Poor: 6,368 lost / 622 new clonotypes (patient-level, descriptive; patient 53 was excluded because paired repertoire data were not available).

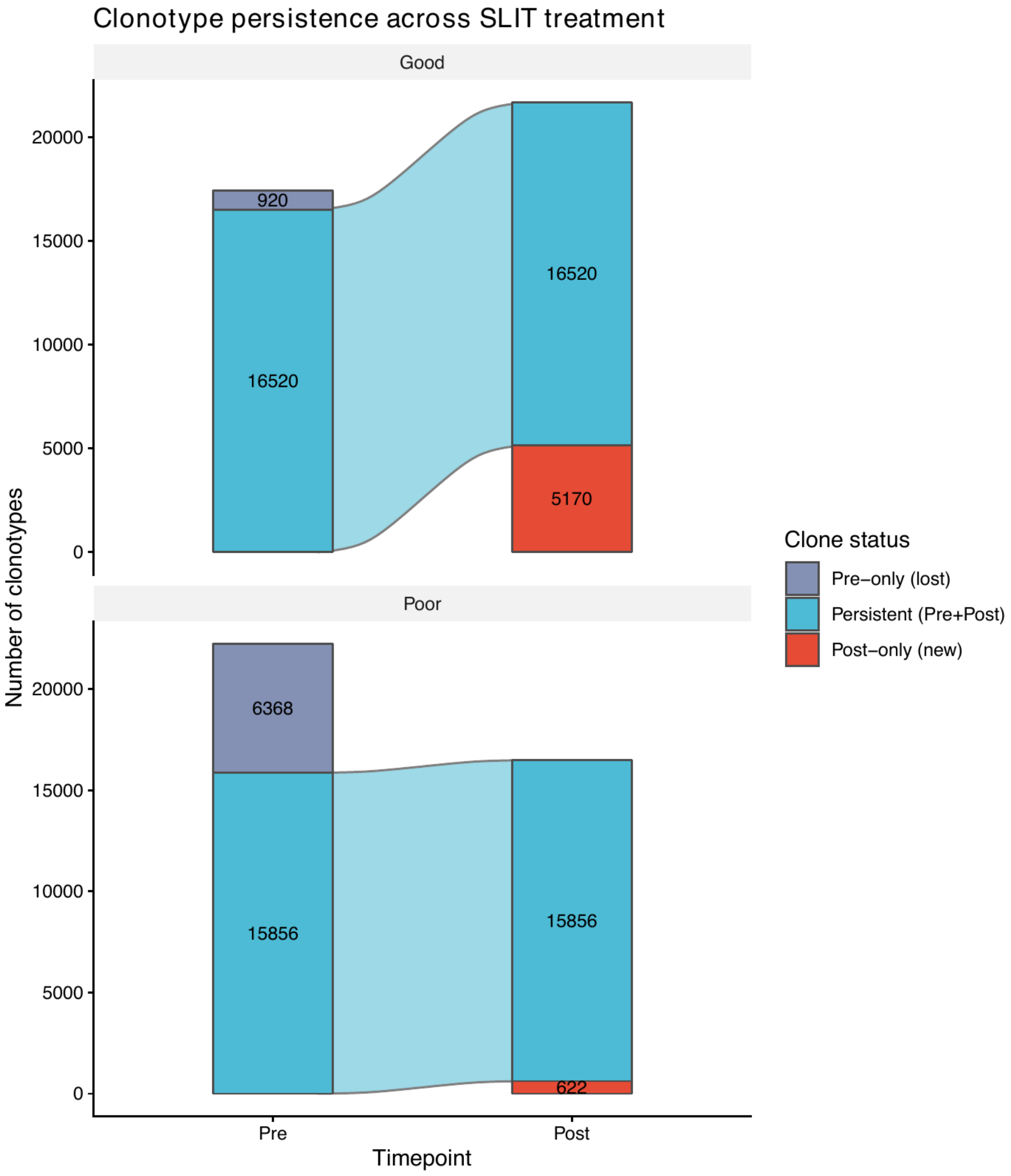

Figure S2. Clone-size change among shared clonotypes before and after SLIT. Points above the diagonal indicate expanded clones; points below indicate contracted clones.

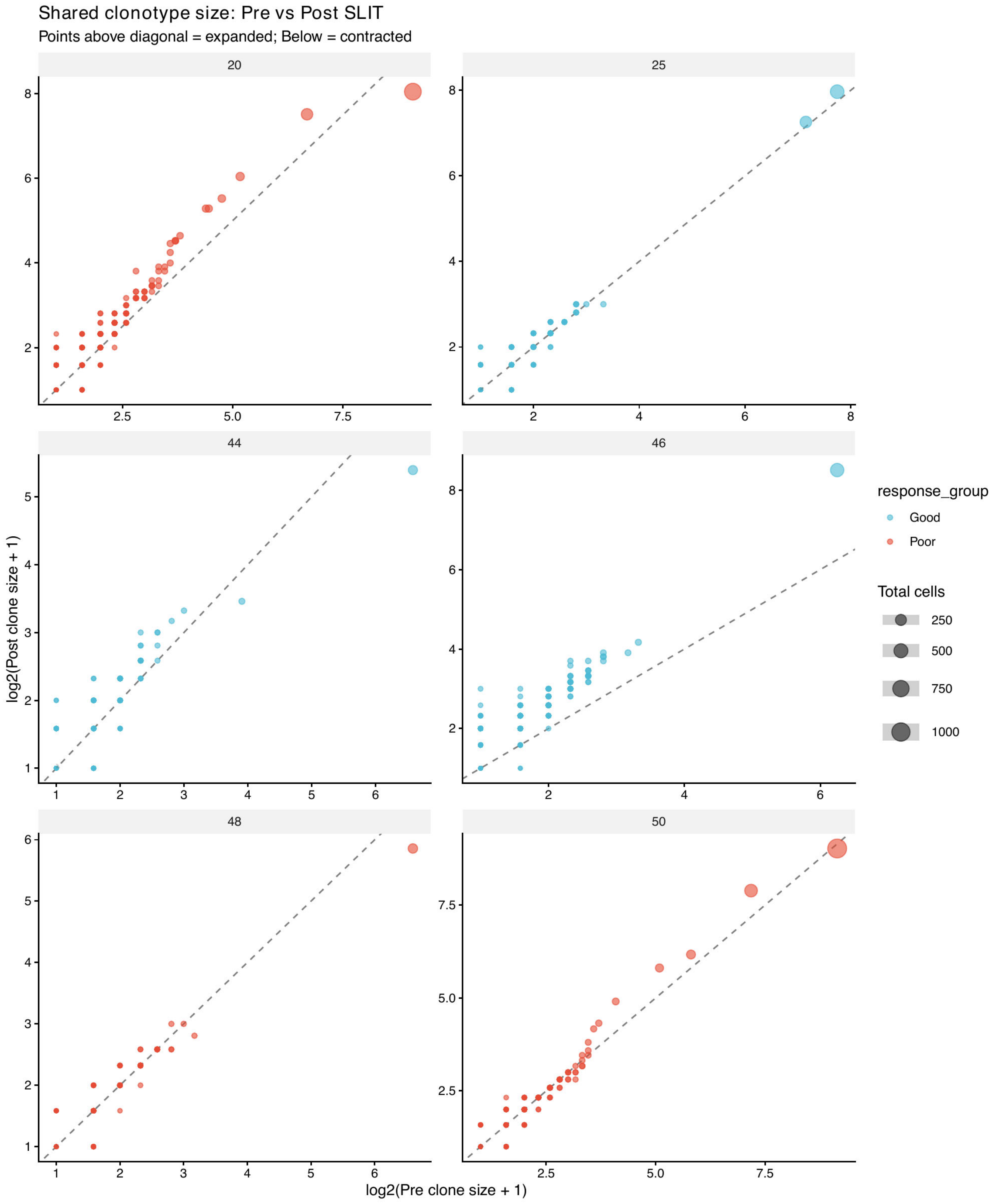

Figure S3. Dominant-state transitions of shared clonotypes in good responders. Percent of Pre clones retained in each Post cell type.

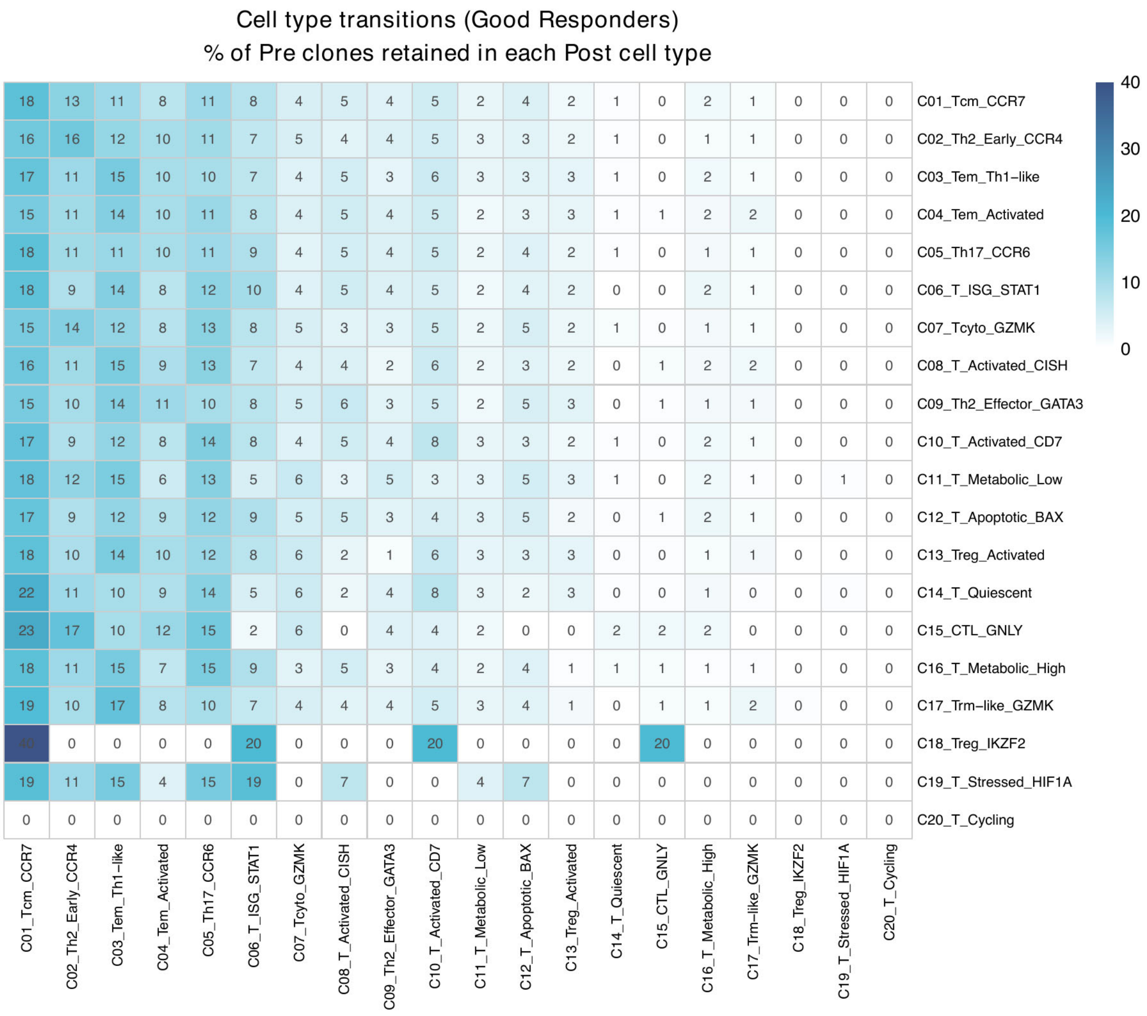

Figure S4. Dominant-state transitions of shared clonotypes in poor responders. Percent of Pre clones retained in each Post cell type.

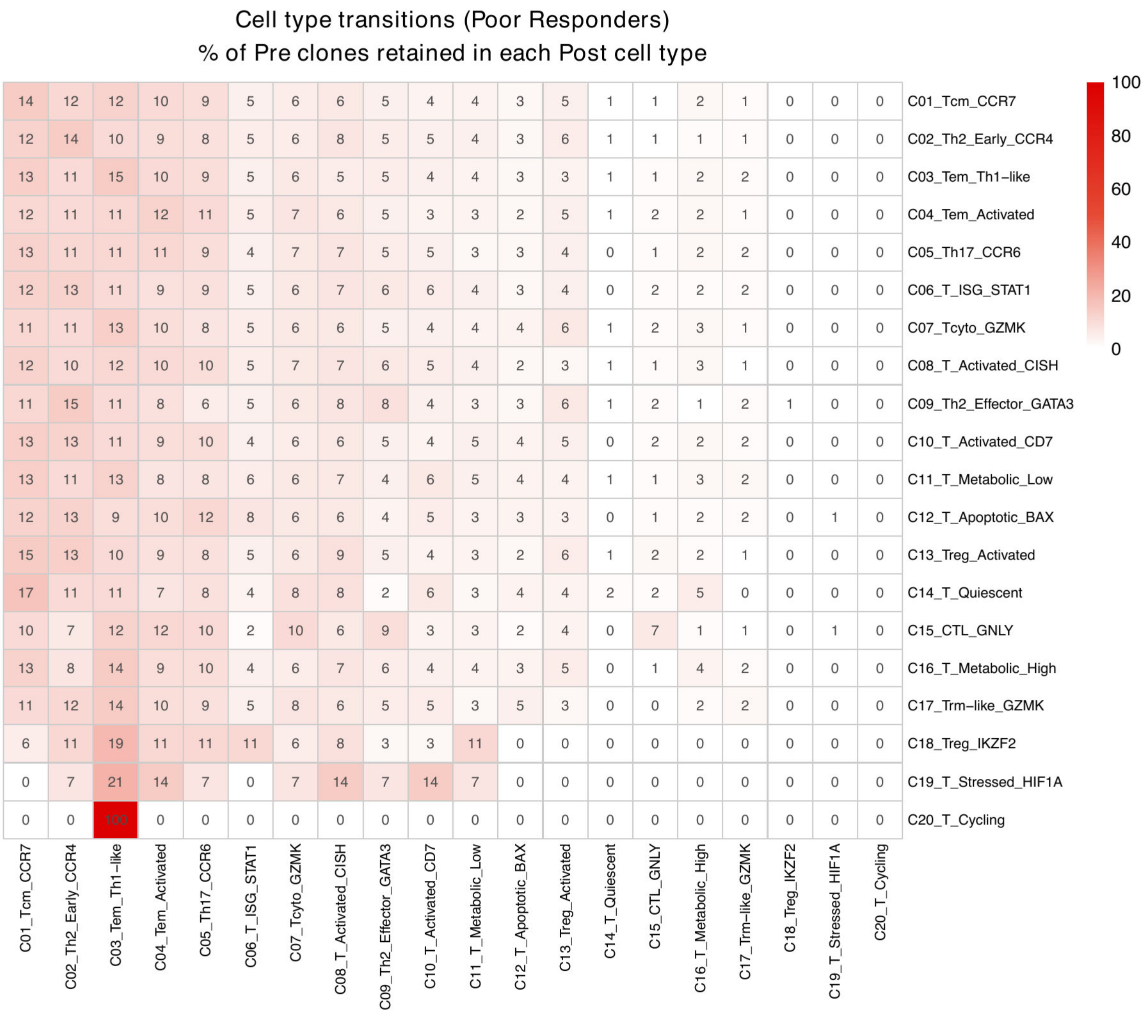

Figure S5. MR core-gene expression across clonotype expansion groups (z-score per gene).

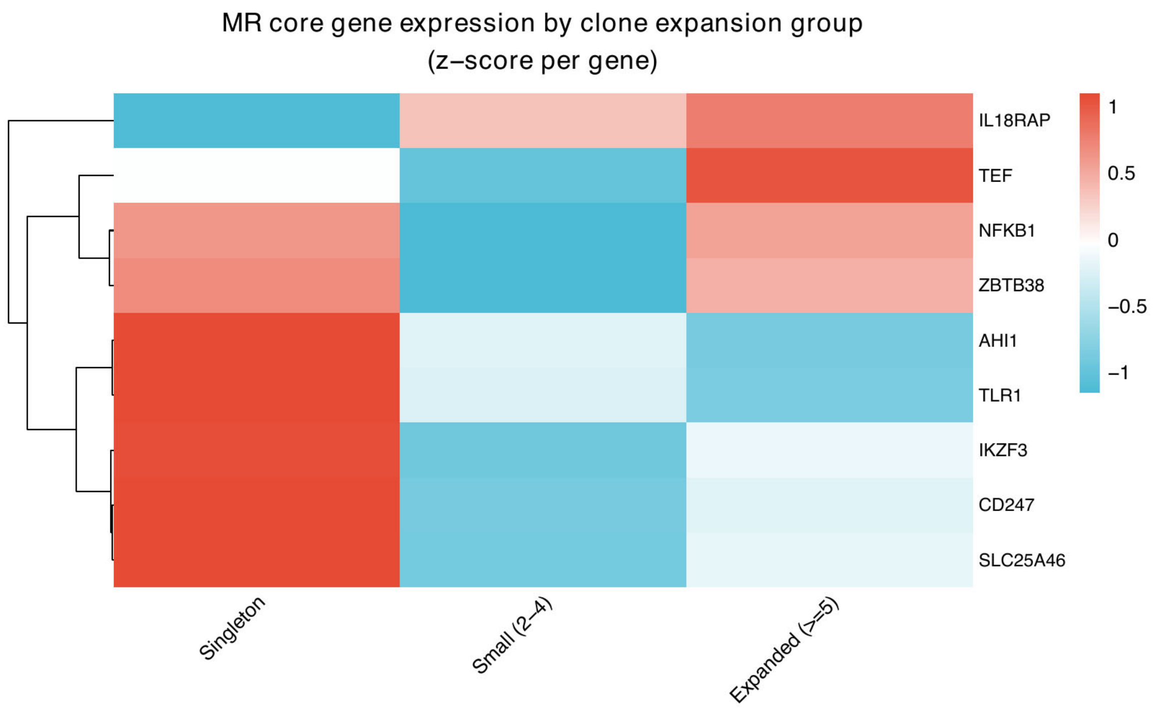

Figure S6. Distribution of representative MR core genes across clone expansion states.

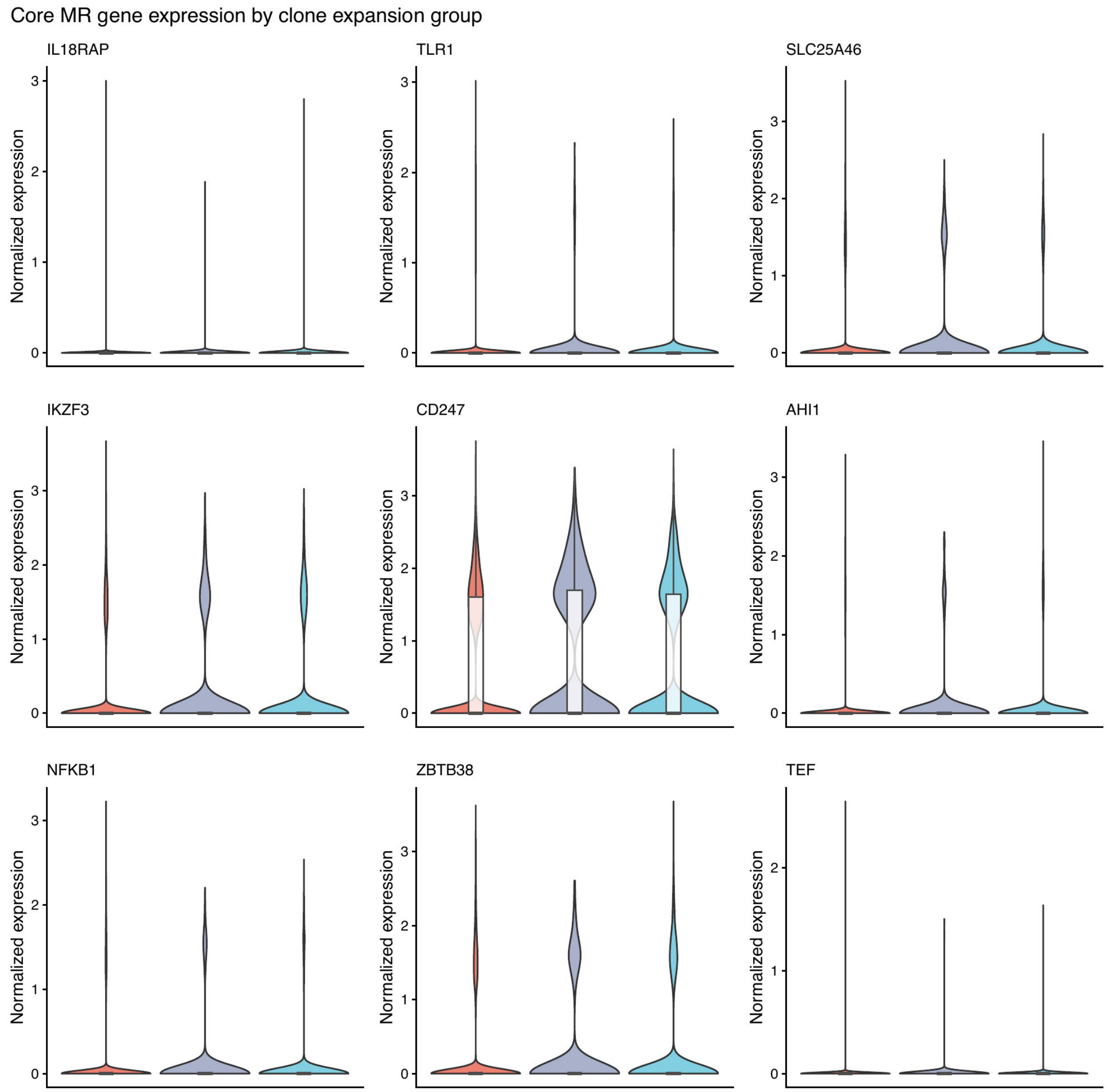

Figure S7. Cell-state composition of newly emergent post-treatment clonotypes.

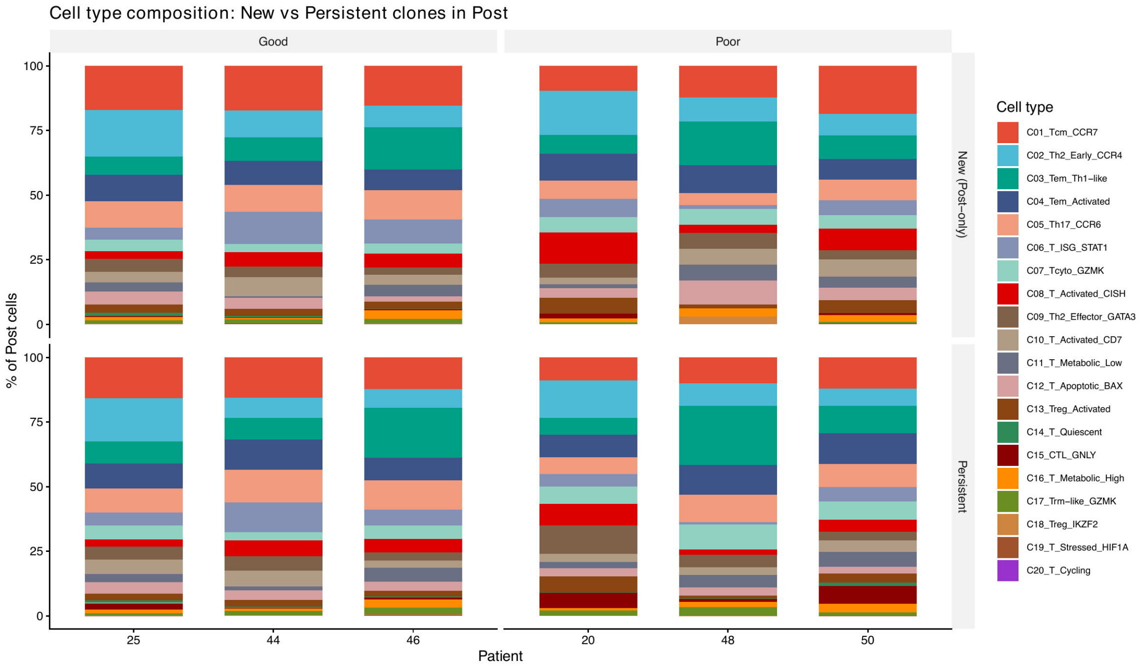

Figure S8. Baseline enrichment of top other-tier MR genes. Red = Poor-enriched, blue = Good-enriched; *FDR < 0.05, **FDR < 0.01, ***FDR < 0.001.

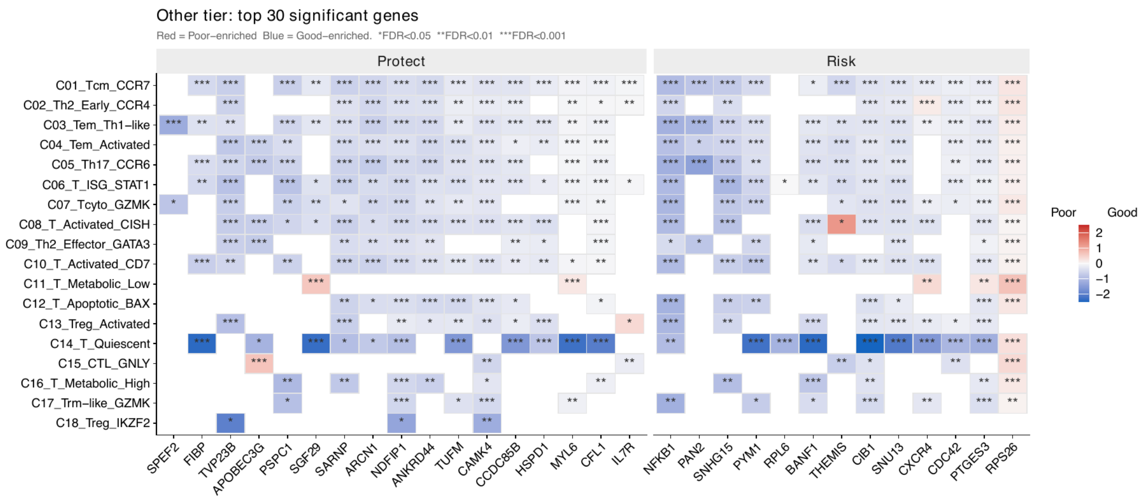

Figure S9. NFKB1 regulon activity across CD4+ T cell subtypes (method: ULM).

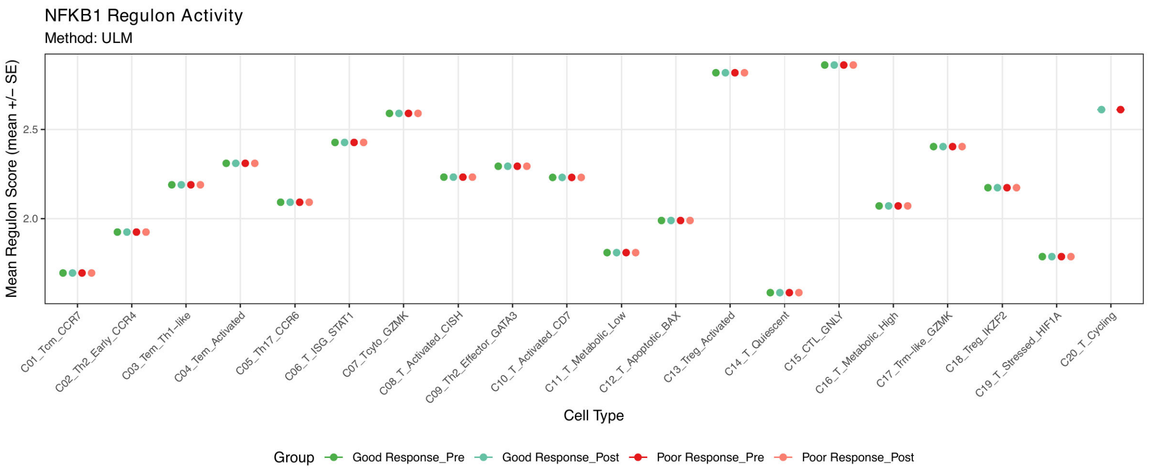

Figure S10. Descriptive comparison between the GSE200107 (SLIT) and GSE273975 (TCM decoction) datasets. X = GSE200107 baseline Poor vs Good (log2 OR); Y = GSE273975 Pre vs Post (log2 Ro/e). The two contrasts are biologically different; GSE273975 (2 patients, 4 libraries, Peiyuan Tongqiao Decoction) serves as a descriptive reference only.

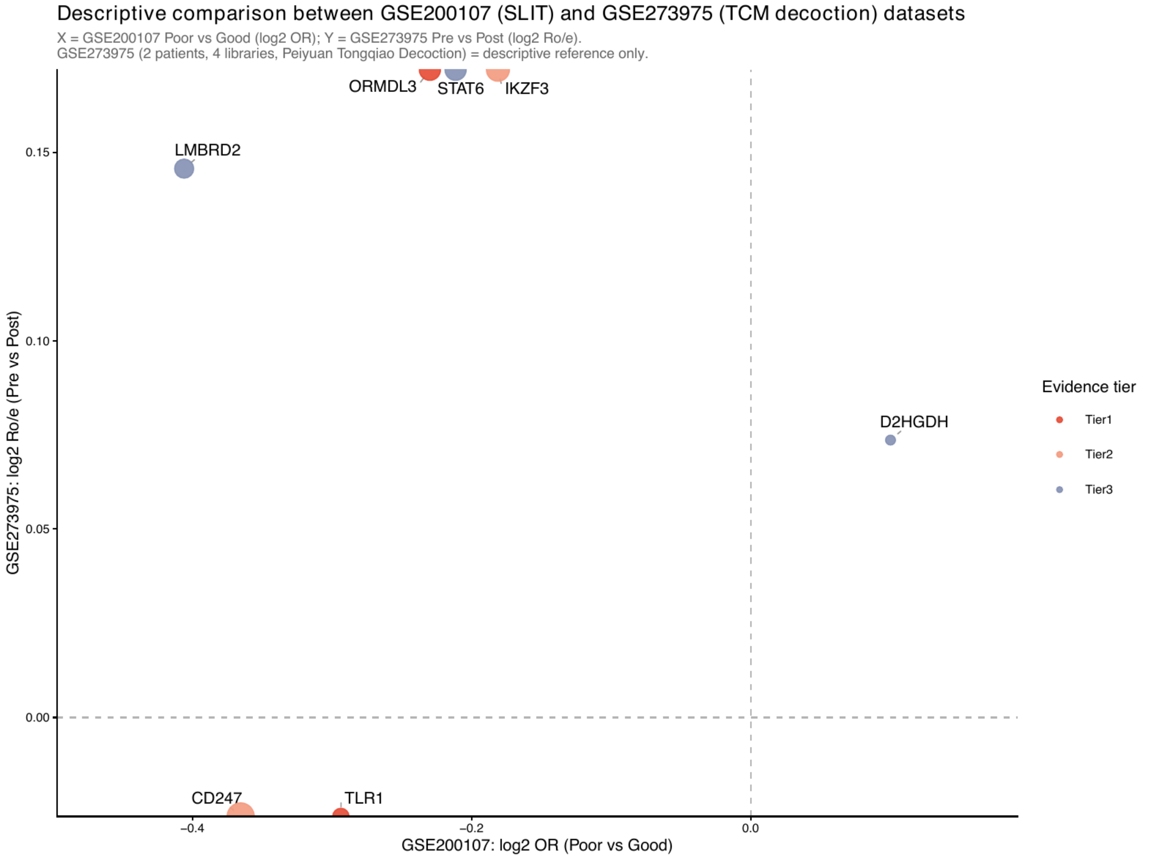

Supplement: Supplementary file 1 [file biomedicines-14-02077-s001.zip › biomedicines-4482591-supplementary/Supplementary_Material_Figures S1-S10.pdf]
